# Acyloxyacyl Hydrolase Protects against Kidney Injury via Inhibition of Tubular CD74-Macrophage Crosstalk

**DOI:** 10.7150/ijbs.91237

**Published:** 2024-05-19

**Authors:** Zhenkai Wu, Bo Deng, Yuqi Shen, Xuezhu Li, Jiaolun Li, Yan Li, Shuai Ma, Yu Pan, Feng Ding

**Affiliations:** 1 Division of Nephrology, Shanghai Ninth People's Hospital, Shanghai Jiao Tong University School of Medicine, Shanghai, China.; 2 Shanghai Key Laboratory of Gut Microecology and Associated Major Diseases Research.

**Keywords:** acyloxyacyl hydrolase, folic acid, fibrosis, PTEC, CD74, scRNA-seq

## Abstract

Renal fibrosis is the common pathway in the progression of chronic kidney disease (CKD). Acyloxyacyl hydrolase (AOAH) is expressed in various phagocytes and is highly expressed in proximal tubular epithelial cells (PTECs). Research shows that AOAH plays a critical role in infections and chronic inflammatory diseases, although its role in kidney injury is unknown. Here, we found that AOAH deletion led to exacerbated kidney injury and fibrosis after folic acid (FA) administration, which was reversed by overexpression of *Aoah* in kidneys. ScRNA-seq revealed that *Aoah^-/-^* mice exhibited increased subpopulation of CD74^+^ PTECs, though the percentage of total PTECs were decreased compared to WT mice after FA treatment. Additionally, exacerbated kidney injury and fibrosis seen in *Aoah^-/-^* mice was attenuated via administration of methyl ester of (S, R)-3-(4-hydroxyphenyl)-4,5-dihydro-5-isoxazole acetic acid (ISO-1), an inhibitor of macrophage inhibition factor (MIF) and CD74 binding. Finally, AOAH expression was found positively correlated with estimated glomerular filtration rate while negatively correlated with the degree of renal fibrosis in kidneys of CKD patients. Thus, our work indicates that AOAH protects against kidney injury and fibrosis by inhibiting renal tubular epithelial cells CD74 signaling pathways. Targeting kidney AOAH represents a promising strategy to prevent renal fibrosis progression.

## Introduction

Chronic kidney disease (CKD) is a global public health problem 1. The prevalence of CKD in people aged 20 years and older is estimated to be 11.1% worldwide 2, 3, and approximately 10.8% (119.5 million people) in China 4. CKD is a prominent risk factor for end-stage renal disease (ESRD), cardiovascular disease and all-cause mortality 5, 6. Thus, exploring the pathogenesis of CKD is required for the development of new treatment.

Interstitial fibrosis is the final common pathogenic process and the histological manifestation of CKD 7, 8. Tubular epithelial cells (TECs) exhibit a dual role in fibrosis, acting as both a victim and a catalyst in the disease progression 9. The injured TECs trigger necroinflammation, partial epithelial-to-mesenchymal transition (EMT) and secretion of various bioactive substances such as proinflammatory cytokines 10, 11 and growth factors 12, which further contributes to kidney injury and fibrosis 9. In addition, the incomplete restoration of TECs following acute kidney injury (AKI) is strongly correlated with the persistence of tubulointerstitial inflammation, proliferation of fibroblasts, and excessive deposition of extracellular matrix 9, 13, promoting the AKI-to-CKD progression 14.

Acyloxyacyl hydrolase (AOAH) is a highly conserved host lipase 15-17. In the kidney, it is expressed in renal proximal tubular epithelial cells (PTECs) as well as immune cells 17. The classic function of AOAH is to inactivate lipopolysaccharide (LPS) by selectively hydrolyzing the secondary acyl chains in the lipid A moiety 18, thus mitigating inflammation and tissue damage 19, 20, promoting the recovery from immune tolerance and the restoration of normal responsiveness 21, 22. In addition, other enzymatic activities of AOAH such as phospholipase and lysophospholipase have been identified 23. Moreover, recent research increasingly shows that AOAH is critical in multiple noninfectious diseases such as allergic asthma 24, 25, psoriasis 26, and chronic pelvic pain 27-29.

Given its importance in inflammation and high expression in the kidney, we investigated the role of AOAH in kidney injury. We discovered that *Aoah^-/-^* mice exhibited exacerbated kidney injury, inflammation and fibrosis in response to different injuries. Single-cell RNA sequencing (scRNA-seq) analysis revealed significantly increased subpopulation of PTECs with expression of CD74, a receptor of macrophage migration inhibitory factor (MIF), in folic acid (FA)-treated *Aoah^-/-^* mice. Additionally, overexpression of *Aoah* or pharmacologic inhibition of CD74 mediated proinflammatory pathway alleviated kidney injury and development of fibrosis in FA-treated *Aoah^-/-^* mice. Furthermore, we performed a preliminary exploration and found that AOAH expression in kidneys was positively correlated with estimated glomerular filtration rate (eGFR) but negatively with the degree of renal fibrosis in CKD patients.

## Methods

Details and additional methods are provided in Supplementary Materials.

### Ethics statement

All animal procedures were conducted in accordance with the ARRIVE (Animal Research: Reporting of In Vivo Experiments) guidelines and with the approval of the Institutional Animal Care and Use Committee (IACUC) of Shanghai Ninth People's Hospital (SH9H-2023-A036-SB). Human tissue samples were provided by tissue bank of Division of Nephrology, Shanghai Ninth People's Hospital, in accordance with the regulations of the tissue bank and local research ethics committee approval (2018-83-T74)*.*

### Animals and models

*Aoah^-/-^* mice were obtained from the National Institutes of Health, USA (R.S. Munford) and were generated as previously described 30. The mutated *Aoah* gene has been backcrossed to C57/BL6J mice for at least 10 generations. Littermate wild-type (WT) mice of the same age and sex produced by *Aoah* heterozygous breeders were used as controls. The details of animal experiments are provided in the Supplementary Materials.

### Single-cell RNA sequencing

ScRNA-seq was performed for kidneys from FA-treated WT and *Aoah^-/-^* mice. The library construction and sequencing were performed by Shanghai Biotechnology Corporation. The details of scRNA-seq are provided in Supplementary Materials.

### Statistics

Unpaired Student's t test (two-tailed) was used to analyze differences between 2 groups. Pearson correlation analysis was used to determine the linear relationship of 2 variables. A *P* value less than 0.05 was considered statistically significant. Data were presented as mean ± standard error of the mean (SEM) and were analyzed using the SPSS software package (SPSS, USA) and the Prism software package (GraphPad, USA).

## Results

### AOAH deficiency exacerbated kidney injury and development of fibrosis

**Figure 1:**
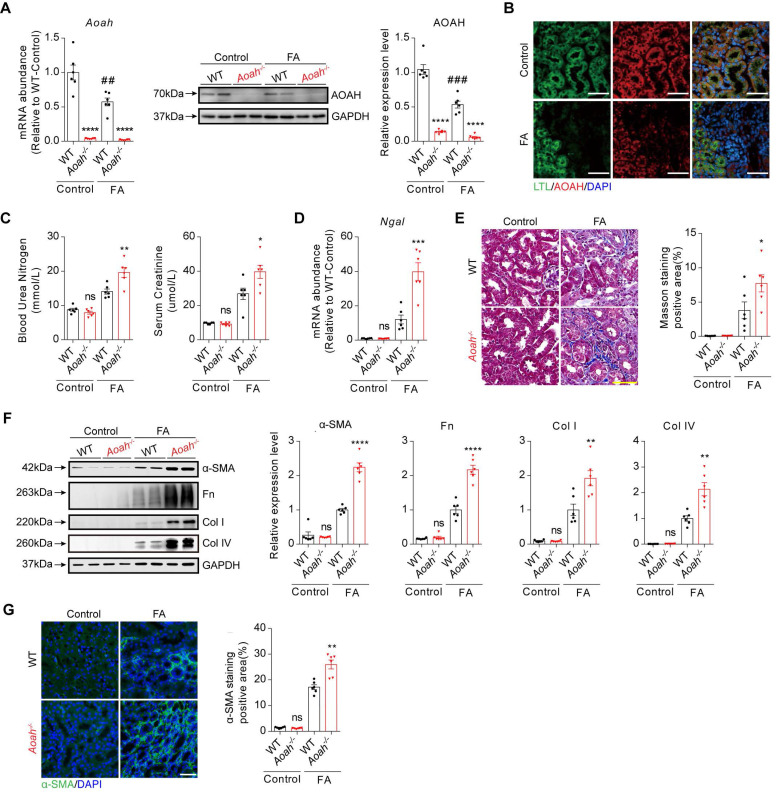
***Aoah^-/-^* mice exhibited more severe kidney injury and fibrosis than WT mice in folic acid (FA)-induced renal fibrosis model.** Male wild-type (WT) and *Aoah^-/-^* mice aged 8-10 weeks intraperitoneally received FA (250 mg/kg) or vehicle and were euthanized 2 weeks later. (A) The expression level of AOAH in kidneys after FA administration was examined by qPCR and western blot. Densitometric analysis of western blots was shown. (B) Immunofluorescence co-staining of AOAH and lotus tetragonolobus lectin (LTL) in kidneys of WT mice after FA treatment. Images of immunofluorescence were taken at 600x magnification. Scale bar, 50 μm. (C) Blood urea nitrogen (BUN) and serum creatinine (SCr) levels on day 14 after FA injection. (D) The *Ngal* mRNA level in whole kidney homogenates after FA administration. (E) Sections were stained for Masson trichrome and positive area was quantified to evaluate the degree of renal fibrosis in mouse kidneys. Scale bar, 50μm. (F) The expression of alpha-smooth muscle actin (α-SMA), fibronectin (Fn), collagen I (Col I) and collagen IV (Col IV) was examined by western blot analysis. Densitometric analysis of western blots was shown. (G) Representative images of immunofluorescence staining of α-SMA. Scale bar, 20μm. The percentage of α-SMA-positive area was analyzed using ImageJ software. Data were presented as mean ± standard error of the mean (SEM). Two-tailed student's t test was used to calculate statistical significance. * Represents comparison between WT and *Aoah^-/-^* mice; # represents comparison within WT mice; *P<0.05, **P<0.01, ***P<0.001, ****P<0.0001; ##*P*<0.01, ###*P*<0.001; ns indicates no significant differences; *n*=6 for all. Similar results were obtained in 3 independent experiments with 6 to 7 mice per group.

To determine the potential role of AOAH in kidney injury, *Aoah^-/-^* mice and corresponding WT mice were treated with FA for 2 weeks. As indicated in Figure 1A, both kidney mRNA and protein levels of AOAH were markedly decreased after FA treatment. Immunofluorescence staining of mouse kidney tissue sections revealed AOAH expression on PTECs, which exhibited a decrease after FA treatment (Figure 1B). Additionally, blood urea nitrogen (BUN) and serum creatinine (SCr) levels were significantly higher in *Aoah^-/-^* mice than in WT mice after FA administration (Figure 1C). *Aoah^-/-^* mice also had more severe kidney injury as indicated by higher neutrophil gelatinase-associated lipocalin (*Ngal*) mRNA levels (Figure 1D). Besides, *Aoah^-/-^* mice exhibited more advanced fibrosis as evidenced by quantitative Masson Trichrome staining (Figure 1E), fibrotic protein markers by immunoblotting (Figure 1F), and quantitative immunofluorescent staining (Figure 1G).

**Figure 2:**
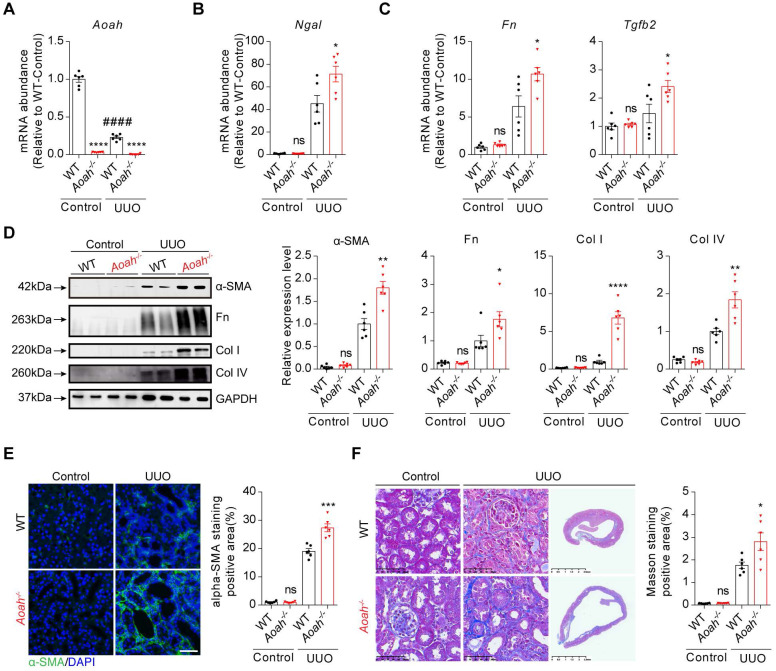
***Aoah^-/-^* mice exhibited more severe kidney injury and fibrosis than WT mice in unilateral ureteral obstruction (UUO)-induced renal fibrosis model.** Male WT and *Aoah^-/-^* mice aged 8-10 weeks were sham-operated or underwent ureter ligation and were euthanized 2 weeks later. (A) *Aoah* mRNA expression after UUO operation was examined by qPCR. (B-C) The mRNA levels of *Ngal*, fibronectin (*Fn*) and transforming growth factor beta 2 (*Tgfb2*) 2 weeks after UUO induction were determined by qPCR. (D) Protein expression of α-SMA, Fn, Col I and Col IV was examined by western blot analysis. Densitometric analysis of western blots was shown. (E) The degree of renal fibrosis was evaluated via immunofluorescence staining of α-SMA and positive area was quantified. Scale bar, 20μm. (F) Representative images of Masson trichrome staining of kidney sections. Scale bar, 50μm. Right panels show kidneys in the full view; Scale bar, 2.5mm. The percentage of interstitial fibrotic area was analyzed using ImageJ. Data were presented as mean ± SEM. Two-tailed student's t test was used to calculate statistical significance. * Represents comparison between WT and *Aoah^-/-^* mice; # represents comparison within WT mice; *P<0.05, **P<0.01, ***P<0.001, ****P<0.0001; ####*P*<0.0001; ns indicates no significant differences; *n*=6 for all. Similar results were obtained in 3 independent experiments with 6 to 7 mice per group.

Unilateral ureteral obstruction (UUO) can induce inflammatory injury and rapid development of progressive tubulointerstitial fibrosis 31. Similar to those seen in FA model, *Aoah* mRNA levels were also markedly decreased 14 days after UUO (Figure 2A). *Aoah^-/-^* mice had more severe kidney injury as indicated by higher *Ngal* mRNA levels (Figure 2B), and more severe renal fibrosis as indicated by increased profibrotic and fibrotic components (Figure 2C-F).

Considering the effect of AOAH on LPS hydrolysis, we also verified the classical role of AOAH in LPS-induced AKI model. As expected, we observed a marked decrease in AOAH expression and a marked increase in BUN and SCr levels in *Aoah^-/-^* mice after LPS injection compared to those in WT mice (Figure S1A-B). The *Ngal* mRNA expression was also significantly increased in mice with lack of *Aoah* (Figure S1C). Additionally, hematoxylin-eosin (HE) staining showed significantly higher tubular injury score in *Aoah^-/-^* mice post LPS administration (Figure S1D).

Taken together, these results suggest that AOAH exhibits a promising effect against renal injury and fibrosis.

### *Aoah^-/-^* mice had significantly increased CD74^+^ PTECs after FA treatment

**Figure 3:**
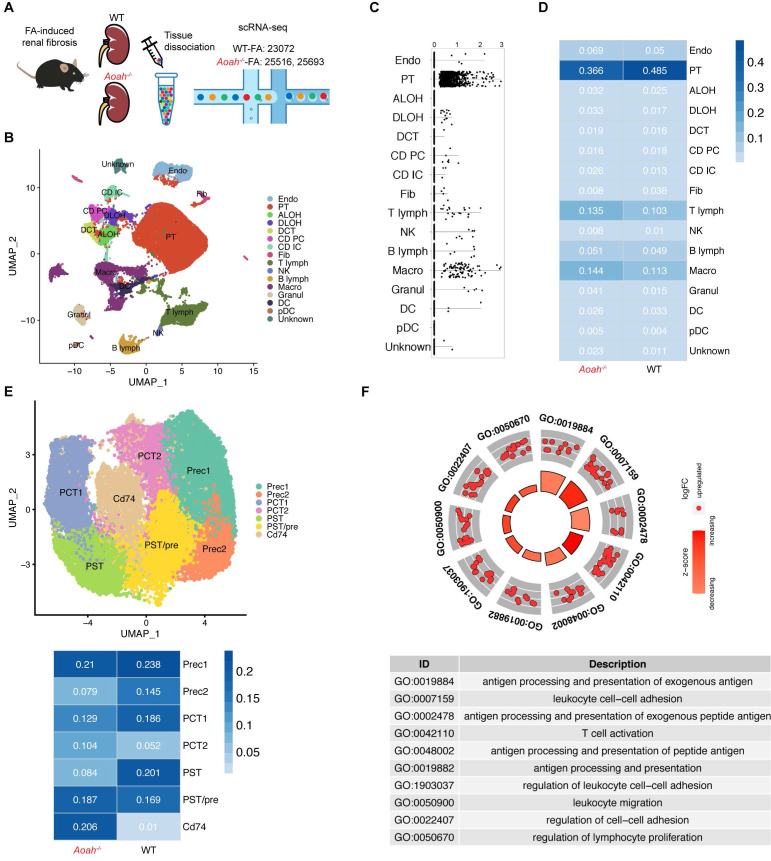
***Aoah* deficiency significantly increased the population of CD74^+^ proximal tubular epithelial cells (PTECs) in FA-induced renal fibrosis.** Single-cell RNA sequencing was performed on kidneys harvested on day 14 from WT mice (*n*=1) and *Aoah^-/-^* mice (*n*=2) after FA induction. (A) Summarized study design and experimental procedure. The sequencing was performed on 10x Genomics Chromium system according to the protocol. (B) UMAP plots showing cell populations within mouse kidney tissues. Assigned clusters include: endothelial (Endo), proximal tubule (PT), ascending limb of Henle's loop (ALOH), descending limb of Henle's loop (DLOH), distal convoluted tubules (DCT), collecting duct principal cell (CD-PC), collecting duct intercalated cell (CD-IC), fibroblast (Fib), T lymphocyte (T lymph), natural killer cell (NK), B lymphocyte (B lymph), macrophage (Macro), granulocyte (Granul), dendritic cell (DC), plasmacytoid dendritic cell (pDC), unknown cells (Unknown). (C) *Aoah* expression in different kidney cell types was shown. (D) Percentages of different cell populations in kidneys of WT and *Aoah^-/-^* mice were analyzed. (E) Subgroup analysis of PT cells was performed and percentages of different PT subpopulations in WT and *Aoah^-/-^* mice were shown. (F) Gene ontology (GO) analysis was used for CD74^+^ PTECs and the top significant terms of GO enrichment analysis were shown.

We further used scRNA-seq to investigate potential mechanisms underlying detrimental effect of *Aoah* deletion in kidney injury after FA administration (Figure 3A). Uniform manifold approximation and projection for dimension reduction (UMAP) diagram revealed high consistency of kidney scRNA-seq samples between *Aoah^-/-^* mice and WT mice (Figure S2). UMAP clustering identified 16 mouse kidney cell clusters (Figure 3B). These cell clusters were assigned to distinct cell types based on known cell-type markers (Figure S3A). As shown in Figure 3C, *Aoah* was mainly expressed in PTECs, macrophages and a few T lymphocytes. Further analysis revealed that *Aoah^-/-^* mice had decreased proportion of PTECs, while displaying an increased proportion of macrophages and T lymphocytes compared to WT mice 2 weeks after FA treatment (Figure 3D).

To determine the potential involvement of macrophages infiltration in FA-induced kidney injury, we treated mice with FA for 24 hours and utilized the kidneys for subsequent analysis. There was no significant difference in the levels of BUN and SCr between WT and *Aoah^-/-^* mice (Figure S4A). Additionally, the qPCR analysis showed similar levels of kidney proinflammatory cytokines between WT and *Aoah^-/-^* mice (Figure S4B). Flow cytometry analysis also showed similar increases in leukocytes, macrophages and neutrophils in WT mice and *Aoah^-/-^* mice (Figure S4C). The initial similarity in immune cell numbers and proinflammatory cytokine expression suggested that the differences observed at later stage may be attributed to variations in PTECs injury.

**Figure 4:**
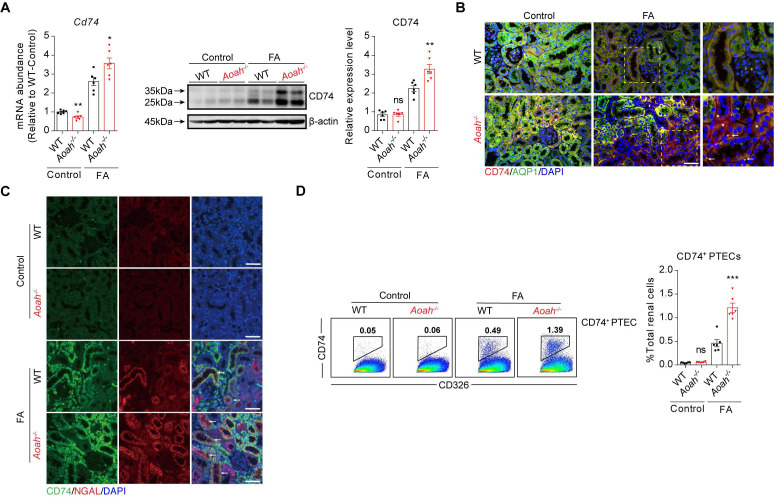
***Aoah* deficiency significantly increased kidney CD74 expression in FA-induced renal fibrosis.** Male WT and *Aoah^-/-^* mice aged 8-10 weeks intraperitoneally received FA (250 mg/kg) or vehicle and were euthanized 2 weeks later. (A) The mRNA and protein levels of CD74 in kidneys after FA administration were examined by qPCR and western blot. Densitometric analysis of western blots was shown. (B) The immunofluorescence double staining of CD74 and aquaporin1 (AQP1) in mice kidney. Right panels are magnification of hatched boxes. Arrows denote CD74^+^ immune cells. Asterisks represent CD74^+^ PTECs. Scale bar, 20μm. (C) The immunofluorescence staining of CD74 and NGAL in mice kidney. Arrows represent CD74^+^ PTECs co-express NGAL. Scale bar, 20μm. (D) Proportion of CD74^+^ PTECs in kidney-derived single-cell suspensions. CD326^+^CD74^+^ cells were considered CD74^+^ PTECs. Data were presented as mean ± SEM. Two-tailed student's t test was used to calculate statistical significance. * Represents comparison between WT and *Aoah^-/-^* mice; *P<0.05, **P<0.01, ***P<0.001; ns indicates no significant differences; *n*=6 for all. Similar results were obtained in 3 independent experiments with 6 to 7 mice per group.

Subsequently, we examined the potential disparities in subsets of PTECs between *Aoah^-/-^* mice and their WT counterparts. The main cell markers used to identify CD74^+^ PTECs were listed in Figure S3B. Although most PTEC subsets in *Aoah^-/-^* mice were decreased compared to WT mice, the percentage of PTECs with CD74 expression was markedly increased (Figure 3E). GO enrichment analysis revealed that CD74^+^ PTECs may be involved in antigen presentation and leukocyte adhesion (Figure 3F). The upregulation of kidney CD74 mRNA and protein levels in FA-treated *Aoah^-/-^* mice was also confirmed by qPCR and immunoblotting (Figure 4A). Immunofluorescent staining showed higher CD74 staining levels in PTECs in FA-treated *Aoah^-/-^* mice, more pronounced loss of brush border and immune cells infiltration (Figure 4B). The CD74^+^ PTECs also exhibited elevated expression of NGAL, a marker of proximal tubular injury (Figure 4C). Flow cytometry data (Figure 4D) also supported above results.

As AOAH was expressed in both PTECs and macrophages in kidneys, we evaluated whether *Aoah* deletion had similar impact on *Cd74* expression in PTECs and macrophages after FA treatment. As shown in Figure S3D, although *Cd74* mRNA level in PTECs was markedly higher in* Aoah^-/-^* mice than in WT mice, its level in macrophages was relatively comparable between *Aoah^-/-^* mice and WT mice, suggesting that AOAH might selectively downregulate CD74 expression solely in PTECs.

### *Aoah^-/-^* mice exhibited increased recruitment of innate immune cells, which interacted with CD74^+^ PTECs to produce proinflammatory cytokines

**Figure 5:**
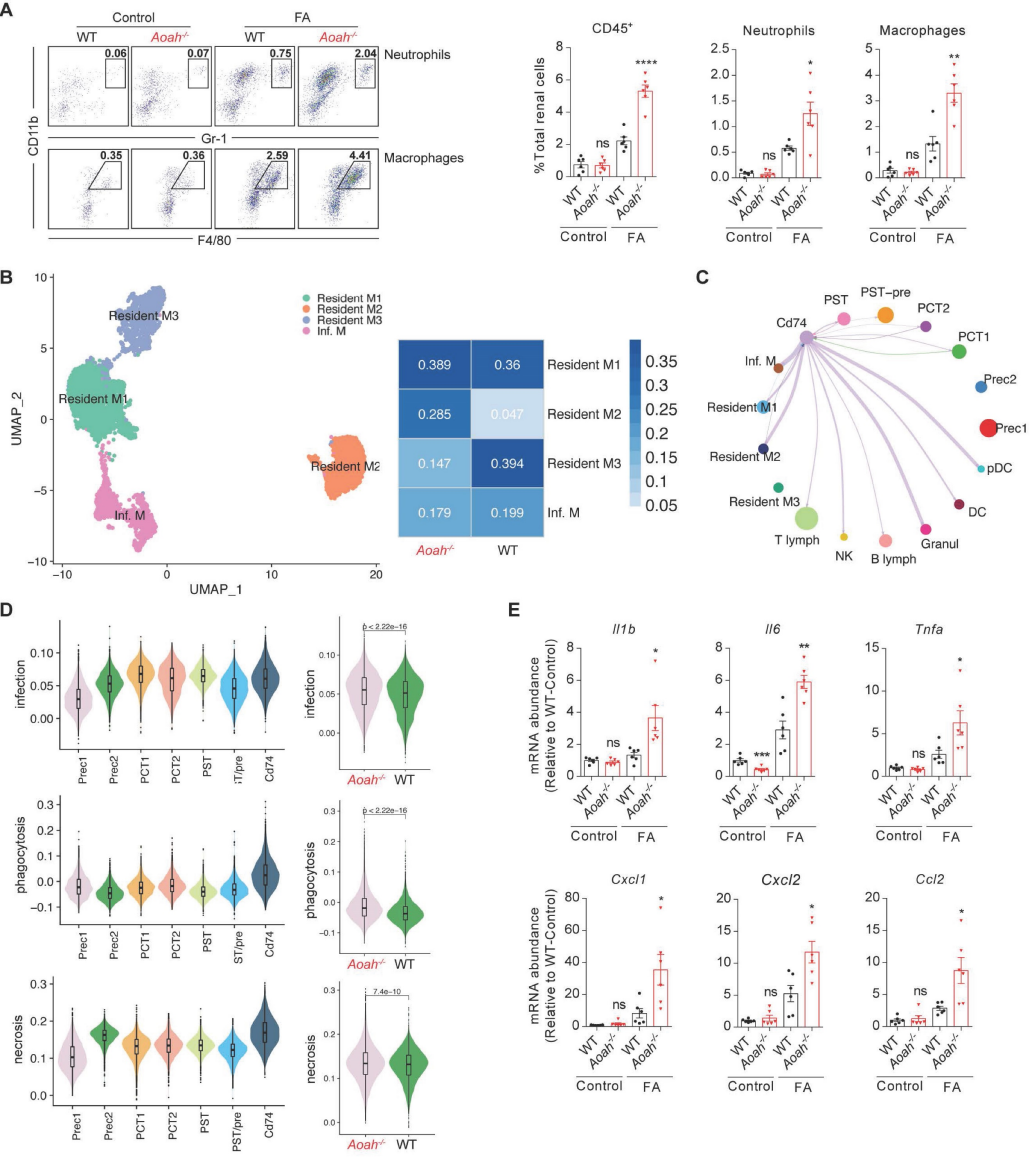
***Aoah* deficiency promoted recruitment of innate immune cells, which interacted with CD74^+^ PTECs to produce proinflammatory cytokines.** Male WT and *Aoah^-/-^* mice aged 8-10 weeks intraperitoneally received FA (250 mg/kg) or vehicle and were euthanized 2 weeks later. (A) Proportion of total leukocytes, neutrophils and macrophages in kidney-derived single-cell suspensions. CD45^+^ cells were considered total leukocytes; CD11b^+^Gr-1^+^ cells were considered neutrophils; CD11b^+^F4/80^+^ cells were considered macrophages. Data were presented as mean ± SEM. Two-tailed student's t test was used to calculate statistical significance. * Represents comparison between WT and *Aoah^-/-^* mice; *P<0.05, **P<0.01, ****P<0.0001; ns indicates no significant differences; *n*=6 for all. Similar results were obtained in 3 independent experiments with 6 to 7 mice per group. (B) Subgroup analysis of macrophages was performed and percentages of different macrophages subpopulations in WT and *Aoah^-/-^* mice were shown. (C) Cell-cell communication between CD74^+^ PTECs and immune cells. (D) Inflammation-related functional analysis of PT cell subsets in WT and *Aoah^-/-^* mice was presented in the form of a violin diagram. (E) The mRNA expression of inflammatory cytokines in kidneys of WT and *Aoah^-/-^* mice was examined by qPCR. Data were presented as mean ± SEM. Two-tailed student's t test was used to calculate statistical significance. * Represents comparison between WT and *Aoah^-/-^* mice; *P<0.05, **P<0.01; ns indicates no significant differences; *n*=6 for all. Similar results were obtained in 3 independent experiments with 6 to 7 mice per group.

Recruitment of innate immune cells contributes to the development of renal fibrosis 32-34. ScRNA-seq showed increased proportion of macrophages in *Aoah^-/-^* mice after FA treatment (Figure 3D). By utilizing flow cytometry analysis, we confirmed more pronounced infiltration of total leukocytes, neutrophils and macrophages in *Aoah^-/-^* mice kidneys 14 days after FA treatment (Figure 5A). Furthermore, scRNA-seq identified 4 different macrophage subpopulations: resident M1, M2, M3, and infiltrating macrophages in kidneys of FA-treated mice, with higher proportion of resident M2 macrophages and lower proportion of resident M3 macrophages in *Aoah^-/-^* mice after FA treatment (Figure 5B and Figure S3C). GO analysis showed that the resident M2 macrophages in *Aoah^-/-^* mice were related to several functional enrichment, including leukocyte migration and regulation of inflammatory responses (Figure S5A), while the resident M3 macrophages were involved in ATP metabolism and the maintenance of normal physiological functions of TECs (Figure S5B). Pseudo-time analysis was used to infer differentiation trajectories of resident macrophages (Figure S5C). We postulated that resident M1 macrophages should have differentiated into resident M3 macrophages in FA-induced renal fibrosis but ended up differentiating into M2 subtype due to *Aoah* deletion. GO enrichment analysis suggested that the differentiation conversion of resident macrophages was primarily associated with alteration in energy metabolism (Figure S5D). Therefore, AOAH may exert an influence on the activation status, energy metabolism and differentiation trajectory of macrophages in renal tissues.

As scRNA-seq analysis has suggested that CD74^+^ PTECs may possess immune-modulatory capacity (Figure 4F), we analyzed the cell-cell communication between CD74^+^ PTECs and immune cells, revealing that CD74^+^ PTECs mainly interacted with resident and infiltrating macrophages, granulocytes as well as plasmacytoid dendritic cells (Figure 5C). We also analyzed PTEC subsets and found that CD74^+^ PTECs exhibited remarkably enhanced functional scores in terms of infection, phagocytosis and necrosis among all subgroups, which was even more pronounced in *Aoah^-/-^* mice (Figure 5D). The qPCR results confirmed that proinflammatory cytokines/chemokines expression including *Il1β*, *Il6*, *Tnfα*, C-C motif chemokine ligand 2 (*Ccl2*), C-X-C motif chemokine ligand 1 (*Cxcl1*) and C-X-C motif chemokine ligand 2 (*Cxcl2*) were increased in *Aoah^-/-^* mice (Figure 5E). Additionally, the immune-modulatory properties of CD74^+^ PTECs were verified in vitro. Mouse tubular epithelial cells (mTECs) were transfected with *Cd74* overexpression plasmids or control plasmids, and cultured with RAW 264.7 macrophages. The efficiency of overexpression was verified by qPCR analysis (Figure S6A). We observed a significant increase in mRNA expression of interleukin-1 beta (*Il1β*), interleukin-6 (*Il6*), tumor necrosis factor alpha (*Tnfα*) in macrophages co-cultured with *Cd74*-overexpressing mTECs compared to those co-cultured with control mTECs after 12 hours of LPS stimulation (Figure S6B).

We gained similar findings in both LPS-induced AKI model (Figure S7A and B) and UUO model (Figure S8). These findings suggested that CD74^+^ PTECs were involved in immune crosstalk and may potentially facilitate inflammatory cells infiltration as well as inflammatory cytokines expression.

Taken together, these results imply that *Aoah* deficiency promotes recruitment of innate immune cells, which interacts with CD74^+^ PTECs to produce proinflammatory cytokines.

### Overexpression of *Aoah* in* Aoah^-/-^* mice attenuated the augmented kidney injury induced by FA

**Figure 6:**
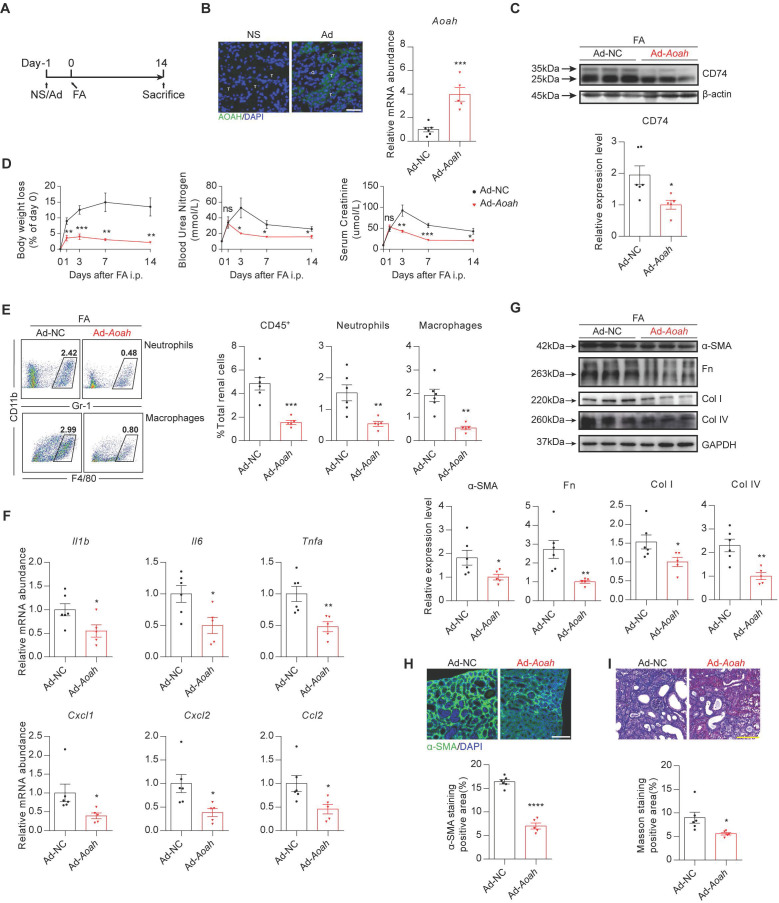
**Renal overexpression of *Aoah* rescued renal fibrosis caused by *Aoah* deficiency in FA-induced renal fibrosis.** Male *Aoah^-/-^* mice aged 8-10 weeks received normal saline (NS), *Aoah* overexpression adenoviruses (Ad-*Aoah*) or control adenoviruses (Ad-NC) respectively by tail vein injection 1 day before FA (250 mg/kg) induction, and were euthanized 2 weeks after FA administration. (A) Summarized design and procedure of the rescue experiment. (B) Infection and rescue efficiency of adenovirus were identified by GFP staining (magnification x400) and qPCR. White ”T” labeled tubules; white “G” labeled glomerulus. Scale bar, 20μm. (C) CD74 expression in kidneys of FA-treated *Aoah^-/-^* mice was examined by western blot after infection. Densitometric analysis of western blots was shown. (D) Body weight, BUN and SCr were recorded at the indicated time points. (E) Proportion of total leukocytes, neutrophils and macrophages in kidney-derived single-cell suspensions. CD45^+^ cells were considered total leukocytes; CD11b^+^Gr-1^+^ cells were considered neutrophils; CD11b^+^F4/80^+^ cells were considered macrophages. (F) The mRNA expression of inflammatory cytokines in kidneys of *Aoah^-/-^* mice that received *Aoah* overexpression adenoviruses or control adenoviruses infection was examined by qPCR. (G) Protein expression of α-SMA, Fn, Col I and Col IV was determined by western blot analysis. Densitometric analysis of western blots was shown. (H) Representative images of kidney sections with α-SMA immunofluorescence staining (magnification x200). Scale bar, 50μm. The positive staining area was quantified via ImageJ. (I) Representative images of Masson trichrome staining and quantification of fibrotic area. Scale bar, 100μm. Data were presented as mean ± SEM. Two-tailed student's t test was used to calculate statistical significance. * Represents comparison between Ad-*Aoah*-infected and Ad-NC-infected *Aoah^-/-^* mice; *P<0.05, **P<0.01, ***P<0.001, ****P<0.0001; ns indicates no significant differences; *n*=3 in NS group, *n*=6 in Ad-NC group, *n*=5 in Ad-*Aoah* group. Similar results were obtained in 3 independent experiments with 3 to 6 mice per group.

We performed a rescue experiment by constructing an adenovirus (Ad) expressing *Aoah* to further verify the effects of AOAH in FA-induced kidney injury. First, HEK-293T infected with* Aoah* adenovirus expression vector was verified by western blot (Figure S9). Then *Aoah^-/-^* mice were treated with saline, control adenoviruse, or *Aoah* overexpression adenoviruse respectively by tail vein injection at day -1 and subsequently treated with FA at day 0 (Figure 6A). Infection efficiency in kidneys was confirmed by GFP staining and qPCR analysis (Figure 6B). Notably, overexpression of *Aoah* resulted in decreased CD74 expression (Figure 6C). Furthermore, *Aoah* overexpression attenuated body weight loss and renal impairment (Figure 6D), resulting in less infiltration of kidney immune cells (Figure 6E) and expression of proinflammatory cytokines/chemokines (Figure 6F), as well as reduction in renal fibrosis (Figure 6G-I).

### Inhibition of CD74 pathway with methyl ester of (S, R)-3-(4-hydroxyphenyl)-4,5-dihydro-5-isoxazole acetic acid (ISO-1) attenuated FA-induced kidney injury

**Figure 7:**
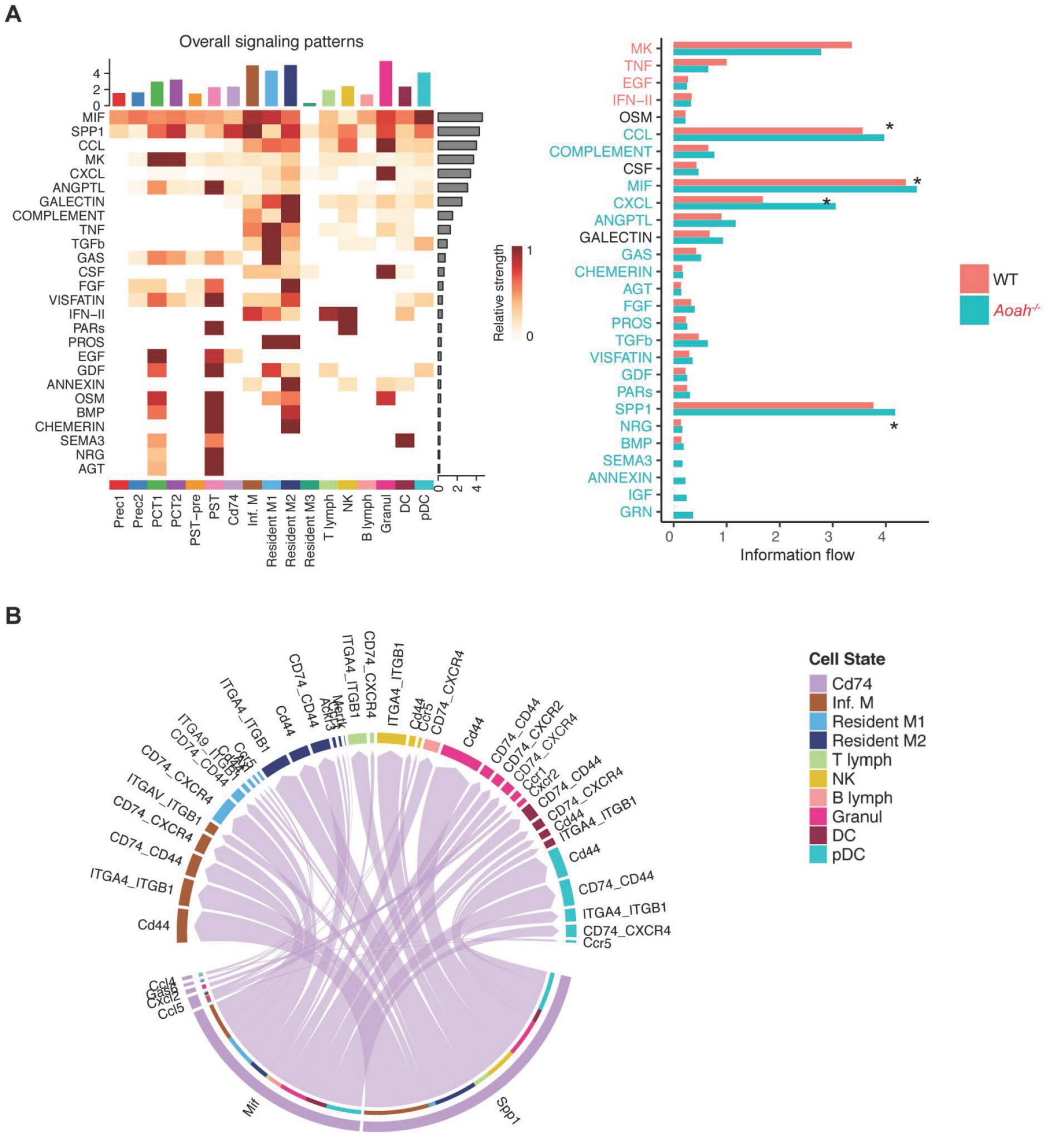
**CD74^+^ PTECs may contribute to renal inflammation and fibrosis by secreting or activating relative ligands.** (A) Differences of cell-cell interaction pathway and receptor-ligand interaction between PT subpopulations and immune cells were analyzed using CellChat. (B) Receptor-ligand pairs were used to infer communication between CD74^+^ PTECs and immune cells.

Further scRNA-seq analysis revealed that macrophage migration inhibitory factor (MIF), secreted phosphoprotein 1 (SPP1), C-C motif chemokine ligand (CCL) family, C-X-C motif ligand (CXCL) family were predominant ligands potentially involved in activating the CD74 pathway to exacerbate inflammation in *Aoah^-/-^* mice after FA administration (Figure 7A). Elevated expression levels of some ligands were verified by qPCR analysis (Figure S10). As shown in Figure 7B, the crosstalk between CD74^+^ PTECs and immune cells was mainly enriched in MIF and SPP1 signaling pathway. Thus, it can be inferred that CD74^+^ PTECs may contribute to renal inflammation and fibrosis by secreting or activating relative ligands.

**Figure 8:**
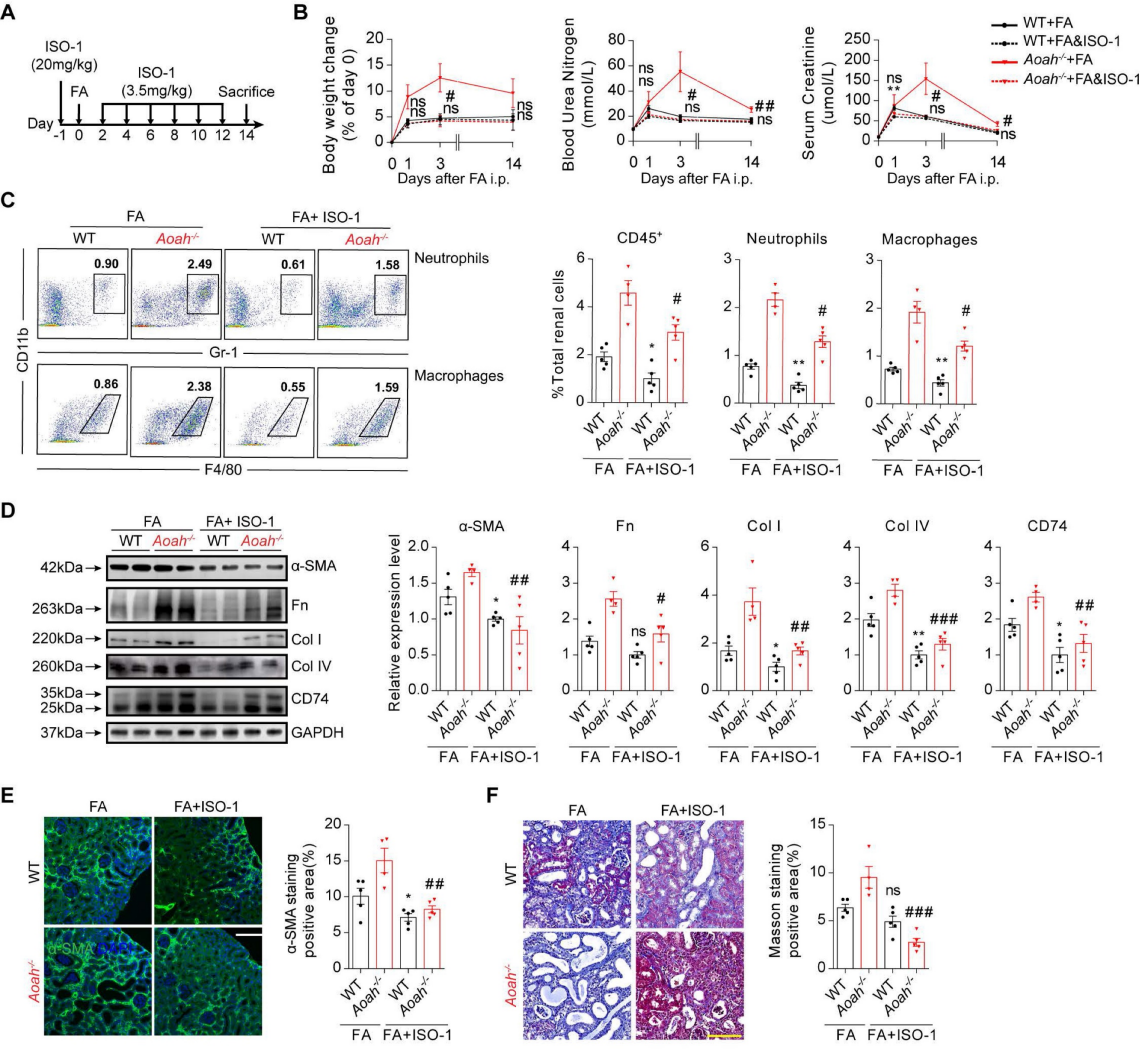
** Exogenous inhibition of CD74 pathway with methyl ester of (S, R)-3-(4-hydroxyphenyl)-4,5-dihydro-5-isoxazole acetic acid (ISO-1) alleviated FA-induced kidney injury and fibrosis.** Male WT mice or *Aoah^-/-^* mice aged 8-10 weeks were randomly assigned to the ISO-1 treatment group or the control group. For the ISO-1 treatment group, mice were given a single dose of ISO-1 (20mg/kg) 1 day before FA induction, and were administered with ISO-1 (3.5mg/kg) every 2 days after. Mice that received an equal volume of vehicle were used as controls. All mice intraperitoneally received FA (250 mg/kg) and were euthanized 2 weeks later. (A) Summarized procedure of the experimental design. (B) Body weight, BUN and SCr were recorded at the indicated time points. (C) Proportion of total leukocytes, neutrophils and macrophages in kidney-derived single-cell suspensions. CD45^+^ cells were considered total leukocytes; CD11b^+^Gr-1^+^ cells were considered neutrophils; CD11b^+^F4/80^+^ cells were considered macrophages. (D) Protein expression of fibrotic components (α-SMA, Fn, Col I and Col IV) and CD74 in kidneys was determined by western blot analysis. Densitometric analysis of western blots was shown. (E) Representative images of kidney sections with α-SMA immunofluorescence staining (magnification x200). Scale bar, 50μm. The positive staining area was quantified using ImageJ. (F) Representative images of Masson trichrome staining and quantification of fibrotic area. Scale bar, 100μm. Data were presented as mean ± SEM. Two-tailed student's t test was used to calculate statistical significance. * Represents comparison between WT mice groups; # represents comparison between *Aoah^-/-^* mice groups. *P<0.05, **P<0.01; #*P*<0.05, ##*P*<0.01, ###*P*<0.001; ns indicates no significant differences; *n*=5 for all, except for ISO-1-treated *Aoah^-/-^* mice group at day 14 (*n*=4). Similar results were obtained in 3 independent experiments with 5 to 6 mice per group.

ISO-1 is the first MIF inhibitor 35, 36, which can bind the MIF tautomerase active site and block the interaction between MIF and CD74 35, 37. Mice in the experimental group were pretreated with ISO-1 (20mg/kg) 1 day before FA injection and thereafter given an injection of ISO-1 (3.5mg/kg) every other day (Figure 8A). ISO-1 treatment led to less loss of body weight, lower BUN and SCr in *Aoah^-/-^* mice (Figure 8B). Flow cytometry analysis indicated that ISO-1 treatment effectively suppressed immune cells infiltration in the kidneys of both *Aoah^-/-^* mice and WT mice (Figure 8C). Additionally, ISO-1 treatment decreased renal CD74 expression in both *Aoah^-/-^* mice and WT mice (Figure 8D). More supporting evidences that ISO-1 alleviated renal fibrosis were provided by western blot (Figure 8D), immunofluorescence staining of α-SMA (Figure 8E) and Masson trichrome staining (Figure 8F).

### The expression of AOAH and CD74 in renal biopsies from patients with CKD

**Figure 9:**
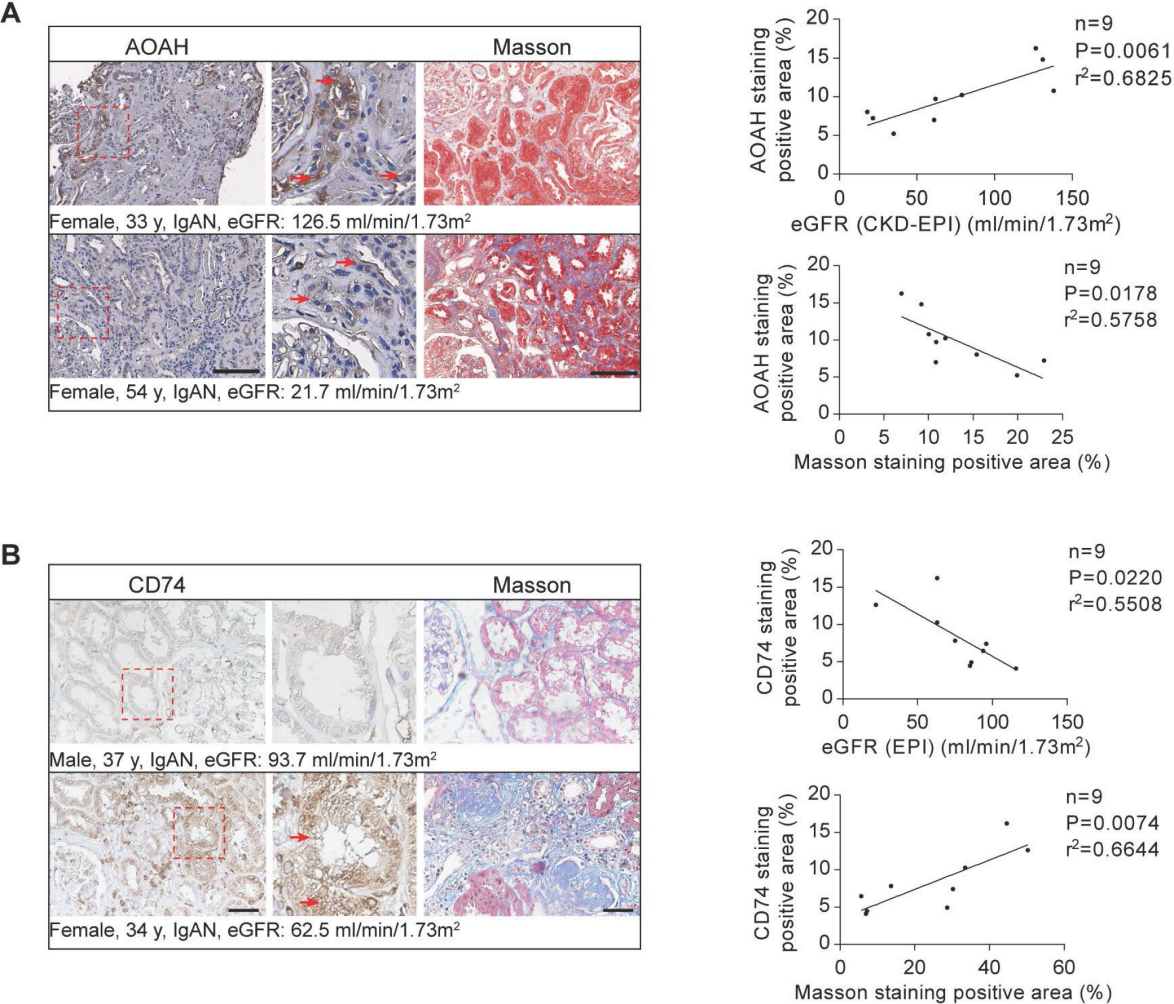
** AOAH and CD74 expression in human CKD biopsies and correlation with disease progression.** Immunohistochemistry and Masson's trichrome staining of 3μm-thick sections were used to analyze the expression of AOAH, CD74 and the fibrotic area in human kidney tissues. Middle panels are magnification of hatched boxes. Arrows denote staining positive renal tubules. (A) The relationship between AOAH expression level in kidneys of CKD patients and estimated glomerular filtration rate (eGFR) (*n*=9; *r^2^*=0.6825; *P*=0.0061) was determined by using linear regression analysis, as was the relationship between AOAH expression level in kidneys of CKD patients and the degree of renal fibrosis (*n*=9; *r^2^*=0.5758; *P*=0.0178). Scale bar, 100 μm. (B) A linear regression analysis was performed to determine the relationship between CD74 expression in kidneys of CKD patients and eGFR (*n*=9; *r^2^*=0.5508; *P*=0.0220), as was the relationship between renal CD74 expression level and the degree of fibrosis (*n*=9; *r^2^*=0.6644; *P*=0.0074). Scale bar, 20 μm.

Previous research has demonstrated highest AOAH expression and activity levels in mouse kidneys 17. However, its expression in human kidneys has not been previously investigated. Therefore, a preliminary study on a small sample of CKD patient kidney biopsies was conducted. Immunohistochemical staining determined that AOAH was primarily expressed in TECs in the kidney biopsy tissue of CKD patients (Figure 9A). We further determined that AOAH expression was positively correlated with eGFR but negatively correlated with the degree of renal fibrosis (Figure 9A). Additionally, we observed a negative correlation between CD74 expression and eGFR levels in CKD patients from the Nephroseq database (nephroseq.org), as depicted in Figure S11. This finding was further supported by immunohistochemistry analysis of kidney biopsies obtained from CKD patients (Figure 9B). Furthermore, our results demonstrated a positive association between CD74 expression and renal fibrosis (Figure 9B).

## Discussion

In the current study, we investigated the potential role of kidney AOAH in response to kidney injury. The major findings are as follows: 1. AOAH deletion exacerbated kidney injury in association with increased inflammation and fibrosis in different kidney injury models; 2. ScRNA-seq revealed that AOAH expression was predominantly observed in PTECs, and a CD74^+^ PTECs subpopulation was selectively expanded in FA-treated *Aoah^-/-^* mice; 3. ScRNA-seq analysis further revealed that CD74-expressing PTECs may contribute to the augmented kidney injury in FA-treated *Aoah^-/-^* mice through activation of the CD74-MIF proinflammatory pathway; 4. Either upregulating *Aoah* or inhibiting CD74-MIF interaction led to ameliorated kidney injury and fibrosis in *Aoah^-/-^* mice after FA treatment; 5. AOAH expression was positively correlated with eGFR but negatively with the degree of renal fibrosis in CKD patients.

AOAH is a highly conserved host lipase originally discovered in phagocytes that selectively hydrolyzes acyloxyacyl bonds in the lipid A moiety, hence detoxifying LPS 15, 18. Feulner *et al*. 17 confirmed AOAH expression in renal cortical tubules and observed that TECs were capable of synthesizing and secreting AOAH into urine, thereby providing protection against urinary tract infections. Additionally, recent research has found high expression level of AOAH in human psoriatic skin lesions 26. However, the expression of AOAH in human kidney tissues has not been previously documented. In this study, we determined the expression of AOAH in human kidneys and established a positive relationship between AOAH expression and eGFR, and showed a negative relationship between AOAH expression and the extent of renal fibrosis. Larger sample size and other biological samples such as blood and urine might help us to illustrate the role of AOAH in human kidneys. We also confirmed that mRNA and protein levels of AOAH significantly decreased in mouse models of kidney injury. In addition, while previous research of AOAH has often focused on its classical enzymatic activity in the LPS model 19-22, 38, there is limited information available regarding a potential role of AOAH in non-microbial organ fibrosis. Here we present evidences for the first time that AOAH exerts a protective effect against renal fibrosis independently of acyloxyacyl hydrolase activity.

CD74, a type II transmembrane glycoprotein, acts as a regulator for protein trafficking and functions as a cell membrane receptor for MIF, d-dopachrome tautomerase and bacterial proteins 35, 39-41. The majority of CD74 protein were present intracellularly, while only a mere fraction (2-5%) can be found on the cell surface 40, 42. Limited data exists regarding CD74 expression in renal cells. Although CD74 is expressed at low baseline levels, abnormal glucose levels and inflammatory cytokines can increase its expression in TECs 36, 42. The data obtained from the Nephroseq database (nephroseq.org) revealed that CD74 expression was negatively correlated with eGFR in patients with IgA nephropathy, focal segmental glomerulosclerosis and other types of CKD. Previous research has reported an upregulation of CD74 expression in both clinical and experimental cases of diabetic nephropathy 42. Moreover, the expression of certain CD74 ligands such as MIF, is also upregulated in kidney disease, which stimulates the expression of inflammatory mediators in TECs and podocytes and promotes inflammatory cells infiltration by interacting with CD74^42^-^45^. However, little evidence exists concerning the involvement of CD74 signaling machinery in renal fibrosis. Recently, researchers discovered that CD74 promotes cyst growth and renal fibrosis in autosomal dominant polycystic kidney disease through MIF/CD74 axis 46. Here, we have demonstrated increased CD74^+^ PTECs as well as elevated CD74 expression in FA-induced renal injury model with the lack of *Aoah*, and suggested that AOAH plays a protective role in renal fibrosis by inhibiting CD74 signaling pathways.

Previous research has shown the therapeutic potential of AOAH in the LPS model. Shao *et al.* 20 found that LPS induced significant enlargement of liver and promoted leukocytes infiltration in *Aoah^-/-^* mice, while recombinant AOAH restored LPS deacylating ability and prevented LPS-induced hepatotoxicity. Liu *et al.* 47 showed that upregulated AOAH expression by overexpression of heat shock protein 12A could reduce cytosolic LPS content, inhibit caspase-11-mediated pyroptosis and protect against LPS-induced liver injury. Here, we found that overexpression of *Aoah* exhibited a potent rescuing effect, which significantly alleviates weight loss and renal impairment, and reduces renal inflammation and fibrosis. Taken together, these findings suggest that AOAH holds promising therapeutic potential for clinical treatment.

We acknowledge that our research may have some limitations. We employed global *Aoah* knockout mice rather than kidney proximal tubule-specific* Aoah* knockout mice. Although the unique role of AOAH in the kidneys has been determined through scRNA-seq, conditional knockout mice and transgenic mice may provide more valuable insights into potential biological mechanism of AOAH in renal injury. Additionally, we currently do not know whether macrophage-derived AOAH are responsible for the worse injury, inflammation and fibrosis in the FA and UUO models. A macrophage deletion strategy or the use of bone marrow chimera approach to separate the role of AOAH in leukocytes compared to intrinsic renal tubular cells may help to address this important point. Our results have suggested that AOAH protected against renal fibrosis by inhibiting CD74 signaling pathway, the precise mechanism underlying the interaction between AOAH and CD74, however, remains unknown. Further work is vital to gain a deeper understanding of the biological mechanism.

In summary, our findings suggest that AOAH plays a novel role in inhibiting renal tubular epithelial cell CD74 signaling pathways and thereby mitigating renal injury and fibrosis through modulation of the tubule-macrophage crosstalk.

## Conclusion

Our study demonstrates that AOAH plays a protective role in renal inflammation and fibrosis by inhibiting renal tubular epithelial cell proinflammatory responses via affecting CD74 signaling pathways. Therefore, targeting kidney AOAH represents a promising strategy to prevent renal fibrosis progression.

## Supplementary Material

Supplementary methods, figures and checklist table.

## Abbreviations

CKD: chronic kidney disease
AOAH: acyloxyacyl hydrolase
PTEC: proximal tubular epithelial cell
FA: folic acid
ISO-1: methyl ester of (S, R)-3-(4-hydroxyphenyl)-4,5-dihydro-5-isoxazole acetic acid
MIF: macrophage inhibition factor
ESRD: end-stage renal disease
TEC: tubular epithelial cell
EMT: epithelial-to-mesenchymal transition
AKI: acute kidney injury
LPS: lipopolysaccharide
scRNA-seq: single-cell RNA sequencing
eGFR: estimated glomerular filtration rate
SEM: standard error of the mean
WT: wild-type
BUN: blood urea nitrogen
SCr: serum creatinine
NGAL: neutrophil gelatinase-associated lipocalin
Fn: fibronectin
α-SMA: αlpha-smooth muscle actin
Col I: collagen I
Col IV: collagen IV
LTL: lotus tetragonolobus lectin
UUO: unilateral ureteral obstruction
UMAP: uniform manifold approximation and projection for dimension reduction
IL1β: interleukin-1 beta
IL6: interleukin-6
TNFα: tumor necrosis factor alpha
CCL: C-C motif chemokine ligand
CCL2: C-C motif chemokine ligand 2
CXCL: C-X-C motif ligand
CXCL1: C-X-C motif chemokine ligand 1
CXCL2: C-X-C motif chemokine ligand 2
SPP1: secreted phosphoprotein 1
Ad: adenovirus
