# Supplementary material for: Acyloxyacyl Hydrolase Protects against Kidney Injury via Inhibition of Tubular CD74-Macrophage Crosstalk
Source: Int J Biol Sci. 2024 May 19;20(8):3061–75. doi: 10.7150/ijbs.91237 (PMC11186370; doi:10.7150/ijbs.91237)
Supplement: Supplementary file 1 — Supplementary methods, figures and checklist table. [file ijbsv20p3061s1.pdf]

Supplementary Methods

Animals and models

All mice were housed in a specific pathogen-free animal facility of Shanghai Ninth People's Hospital. Mice were housed under standard conditions with free access to water and standard laboratory diet. *Aoah*$^{-/-}$ mice and their control littermates (WT mice) were housed in separate cages, with 5-6 mice per cage. All animal procedures were performed in accordance with the guidelines and with the approval of the Institutional Animal Care and Use Committee (IACUC) of Shanghai Ninth People's Hospital, Shanghai Jiaotong University of Medicine. The detailed construction process of experimental animal models was as follows.

Folic acid (FA) induced renal fibrosis model

Male WT mice or *Aoah*$^{-/-}$ mice aged 8-10 weeks weighing approximately 20-24 g were randomly assigned to the control group or the experimental group (n=6 for each group) by cage. For the FA-induced renal fibrosis model, a single dose of folic acid (F7876, Sigma, USA, 250 mg/kg) dissolved in 0.3 M sodium bicarbonate was injected intraperitoneally to each mouse. Mice that received an equal volume of vehicle were used as controls. All mice were euthanized at day 14 after folic acid injection, and blood and kidney samples were collected for further examination.

Unilateral ureteral obstruction (UUO) induced renal fibrosis model

Male WT mice or *Aoah*$^{-/-}$ mice aged 8-10 weeks weighing approximately 20-24 g were randomly assigned to the control group or the experimental group (n=6 for each group) by cage. For the UUO model, mice underwent ligation of the left ureter, and sham-operated mice were used as controls. All mice were euthanized at day 14 after operation, and blood and kidney samples were collected for further examination.

Lipopolysaccharide (LPS) induced acute kidney injury (AKI) model

Male WT mice or *Aoah*$^{-/-}$ mice aged 8-10 weeks weighing approximately 20-24 g were randomly assigned to the control group or the experimental group (n=6 for each group) by cage. For the LPS-induced AKI model, LPS (O111:B4, L4130, Sigma, USA, 10mg/kg) in saline (0.9%) was injected intraperitoneally to each mouse. Mice that received an equal volume of vehicle were used as controls. All mice were euthanized 24 hours after LPS injection, and blood and kidney samples were collected for further examination.

Cell experiment

The mouse tubular epithelial cells (mTECs) were cultured with DMEM/F12 (Gibco, USA) supplemented with 10% FBS (CellCo, China) and 1% penicillin/streptomycin (VivaCell, China). RAW 264.7 cells were cultured in high glucose DMEM (Gibco, USA) supplemented with 10% FBS and 1%

penicillin/streptomycin. All cells were cultured at 37℃ and 5% CO_2 in a sterile incubator. *Cd74* overexpression plasmids and control plasmids were produced by Genomeditech Corp. (Shanghai, China). Lipo8000 (Beyotime, China) was used to transfect *Cd74* overexpression plasmids or control plasmids into mTECs accoding to the manufacturer's instuctions. For co-culture experiment, *Cd74*-overexpressing mTECs or control mTECs were inoculated in the upper chamber, and RAW 264.7 macrophages were inoculated in the lower chamber. RAW 264.7 cells were then received a low-dose of LPS (100ng/ml) to induce M1 polarization. After 12 hours, RAW 264.7 cells were collected for qPCR.

Rescue experiment

Aoah overexpression HEK-293T cells, as well as *Aoah* overexpression adenoviruses (GPAAV-CMV-Mouse-*Aoah*-6xHis-T2A-eGFP-WPRE) and control adenoviruses were produced by Genomeditech Corp. (Shanghai, China). *Aoah*$^{-/-}$ mice aged 8-10 weeks weighing approximately 20-24 g were randomly selected to receive tail vein injection of normal saline (n=3), control adenoviruses (n=6) or *Aoah* overexpression adenoviruses (1e12VG per mouse; n=5). The next day, all mice were treated with folic acid as described above. All mice were euthanized at day 14 after folic acid injection, and blood and kidney samples were collected for further examination.

ISO-1 treatment experiment

ISO-1 was purchased from Selleck Chemicals Company (Shanghai, China). Male WT mice or *Aoah*$^{-/-}$ mice aged 8-10 weeks weighing approximately 20-24 g were randomly assigned to the control group or the experimental group (n=5 for each group) by cage. For ISO-1 treatment, a single dose of ISO-1 (20mg/kg) dissolved in 1% DMSO was injected intraperitoneally to each mouse at day -1. Mice were also treated with ISO-1 (3.5mg/kg) given every other day after folic acid administration. The dosage of ISO-1 was decided with reference to previous studies[1, 2]. Mice that received an equal volume of vehicle were used as controls. All mice were treated with folic acid at day 0 as described above. All mice were euthanized at day 14 after folic acid injection, and blood and kidney samples were collected for further examination.

Single-cell RNA sequencing (scRNA-seq)

Preparation of single cell suspension

The kidney tissues were harvested after cardiac perfusion with cold PBS, then weighed, minced and incubated with 1 mg/mL type IV collagenase and 10μg/mL DNase I (both from Sigma, USA) for 40 minutes at 37℃ shaker. The digested tissue suspensions were then passed through a 40μm cell strainer and washed in PBS with 1% FBS. Erythrocytes were lysed and single cell suspension was acquired. Cells were stained with 0.4% trypan blue solution (Thermo Fisher, USA), and cell viability was was examined by Countess®II

Automated Cell Counter (Thermo Fisher, USA).

ScRNA-seq library construction and sequencing

Single-cell RNA sequencing libraries were prepared using the Chromium Single Cell 3′ v2 Reagent Kit (10x Genomics) according to the manufacture's protocol. Briefly, single cells were encapsulated in oil beads with a unique molecular identifier (UMI) barcode to generate single-cell Gel Beads-in-Emulsion (GEMs). Then cells were lysed and the released RNA was barcoded through reverse transcription in individual GEMs. After the reverse transcription step, cDNAs were amplified, fragmented and used for 3' gene expression library construction. Sequencing of the libraries was performed on a NovaSeq6000 (Illumina) using paired-end 2×150 bp sequencing.

ScRNA-seq data processing

Raw sequencing data were processed following the Chromium's Cell Ranger 3.1.0 pipeline with default parameters. The raw sequencing data (FASTQs files) were aligned to the mouse genome using STAR algorithm. Then gene-barcode matrices containing the barcoded cells and gene expression counts were generated and imported into the Seurat (version 4.2.0) R toolkit for quality control and subsequent analysis. Unless specified, the default parameters were used for all functions. For quality control, cells with detected genes

between 200 to 5000, mitochondrial gene percentages less than 30%, unique gene counts between 200 to 20000 were kept. In the remaining cells, gene expression matrices were log normalized for each cell by the total expression and multiplied this by a scale factor (10000 by default). Principal component analysis (PCA) for dimensional reduction was performed based on the highly variable genes (top 2000). Clusters were then visualized using the Uniform Manifold Approximation and Projection (UMAP) (ArXiv e-prints 1802.03426, 2018). Cell type identification were performed based on the expression of known cell markers[3-5].

Gene set scoring analysis

For gene set scoring analysis, inflammation-related gene signatures and pathways based on the IPA database were compared in proximal tubular cells subpopulation using the “AddModuleScore” function in the Seurat package[6]. The results were shown in the form of violin plots.

GO analysis

Gene Ontology (GO) analysis was performed using the enrichGO function of the clusterProfiler package in R. Biological process and 0.05 *P* value cutoff were chosen[7, 8]. The *P* values were adjusted via the Benjamini–Hochberg method. The results were shown in the form of GOCircle. Bar plot color intensity in the inner circle was based on z-scores, and the height of each bar

indicated each GO term's significance (*P* value).

Cell-cell interaction/communication analysis

Ligand Receptor Pair database CellChatDB was used for the cell-cell interaction/communication analysis through the standard pipeline of the R package CellChat to identify signaling patterns, predict pathways and information flow[9, 10]. The results were shown in the forms of circle plot and bubble plot.

Pseudotime analysis

Pseudotime analysis was performed using the Monocle package 2.16.0 with default parameters to reconstruct cell differentiation trajectories[10, 11]. The obtained gene modules were analyzed and compared through GO analysis to show functional changes during cellular differentiation. The actual gestational time of each cell informed us of the start point of the pseudo-time in the first round of 'orderCells'. 'DDRTree' was applied to reduce dimensions and the visualization functions 'plot_cell_trajectory' were used to plot the minimum spanning tree on cells.

Measurement of BUN and SCr

BUN levels were determined in 5ul of serum using a QuantiChrom^{TM} Urea Assay Kit (DIUR-100, BioAssay Systems, USA) according to the provided

instructions. SCr levels were determined in 30ul of serum using a QuantiChrom^{TM} Creatinine Assay Kit (DICT-500, BioAssay Systems, USA) following the manufacturer's instructions. Each serum sample was tested three times to obtain the average value.

Histology staining

The kidney tissues were fixed with paraformaldehyde (4%), after 48 hours, paraffin-embedded and sliced into 3μm-thick sections, and then processed for hematoxylin-eosin (HE) staining or Masson's Trichrome staining. For HE staining, kidney histological changes were estimated at 10 randomly selected fields (magnification x200) and evaluated in a double-blind fashion. Tubular injury was defined as cellular degeneration and vacuolization, reduction of brush border epithelium, tubular obstruction, and cast formation. Renal tissue damage was scored according to the percentage of damaged tubules: 0, no damage; 1, damage less than 25% of tubular area; 2, damage between 25% and 50% of tubular area; 3, damage between 50% and 75% of tubular area; 4, damage more than 75% of tubular area. Matrix deposition within the interstitium was assessed using Masson's Trichrome stain. Slides were observed under a light microscope (Olympus, Japan) and were finally scanned using a Digital Slide Scanner (KF Bio, China) and viewed with K-Viewer 1.5.5.2 (KF Bio, China). Ten cortical fields (magnification x400) were randomly selected for each kidney slide. The percentage of interstitial fibrotic area was

measured using ImageJ software (ImageJ, NIH).

Immunohistochemistry

Slides of normal kidney tissue and chronic kidney disease from patients were provided by tissue bank of Division of Nephrology, Shanghai Ninth People's Hospital, School of Medicine, Shanghai Jiao Tong University, Shanghai, China. Procedures were in accordance with the regulations of the tissue bank. For immunohistochemistry, tissue sections were deparaffinized and performed heat mediated antigen retrieval with citrate buffer (pH 6.0) for AOAH staining and Tris/EDTA buffer (pH 9.0) for CD74 staining. The next steps were performed using a UltraSensitive™ SP IHC Kit (Maixin Bio, China) following the manufacturer’s protocol. The localization of peroxidase was determined using a DAB kit (Vector, USA). Slides were viewed using a Digital Slide Scanner (KF Bio, China) or a Nikon camera (Nikon, Japan). Immunostaining changes were estimated at 10 randomly selected fields and evaluated in a double-blind fashion. The percentage of positive area after immunohistochemistry was measured using ImageJ software (ImageJ, NIH). The details of the primary antibody used are listed in Table S1.

Immunofluorescence

The kidney tissues were fixed with paraformaldehyde (4%), after 48 hours, paraffin-embedded and sliced into 3μm-thick sections. For

immunofluorescence, tissue sections were deparaffinized and performed heat mediated antigen retrieval with citrate buffer (pH 6.0) or Tris/EDTA buffer (pH 9.0), blocked, incubated with primary antibodies at 4℃ overnight. After washing steps, tissue sections were incubated with the appropriate secondary antibodies for 1 hour at room temperature. The nuclei were stained with DAPI and coverslipped. Immunofluorescence staining images were observed and captured with a Nikon color DS-Ri2 camera using Nikon NIS-elements software (Nikon, Japan). Immunostaining changes were estimated at 10 randomly selected fields and evaluated in a double-blind fashion. The percentage of positive area after immunofluorescence was measured using ImageJ software (ImageJ, NIH). The details of primary and secondary antibodies used are listed in Table S1.

Western blot analysis

The kidney tissues were homogenized in RIPA buffer containing phosphatase inhibitor. Protein concentration was determined using a BCA protein assay kit (Beyotime, China). Equal amounts of sample proteins (10~50μg) mixed with 5x loading buffer were added in 6%~12% SDS/PAGE gels and then transferred to polyvinylidene fluoride membranes. The membranes were blocked with blocking buffer (NCM, China) at room temperature for 30 minutes, and after washing steps, incubated with primary antibodies at 4℃ overnight. After washing steps, the membranes were incubated with the appropriate

secondary antibodies for 1 hour at room temperature. Enhanced chemiluminescent detection was done according to the protocol (NCM, China). Images were acquired with a Amersham Imager 600 (GE Amersham, USA) using the semi-auto exposure mode. Protein expression levels were quantified using ImageJ software (ImageJ, NIH). The details of primary and secondary antibodies used are listed in Table S1.

Quantitative real-time PCR

Total RNA was extracted from mice kidneys using the RNA-simple total RNA isolation kit (DP419, Tiangen, China) according to the provided instructions, and then reversely transcribed to cDNA using a HiScript Ⅲ RT SuperMix for qPCR (R323-01, Vazyme, China). Quantitative real-time PCR was performed using the ChamQ Universal SYBR qPCR Master Mix (Q711-02, Vazyme, China) on Applied Biosystems® QuantStudio™ 6 Flex (Applied Biosystems, USA). The fold induction of the target gene was calculated using the comparative method by normalization to the internal control β-actin. The primer sequences are listed in the Table S2.

Flow cytometry analysis

The kidney tissues were harvested after cardiac perfusion with cold PBS, then weighed, minced and incubated with 1 mg/mL type IV collagenase and DNase I (both from Sigma, USA) for 40 minutes at 37℃ shaker. The digested tissue

suspensions were then passed through a 40μm cell strainer and washed in PBS. Erythrocytes were lysed and single cell suspension was acquired. Apart from one blank control, cells were incubated with propidium iodide (BD, USA) for 10 min before fixation. After blocking nonspecific Fc binding with anti-mouse CD16/32 antibody (Biolegend, USA), kidney cell suspensions were incubated with anti-mouse CD45-APC/Cy7 (Biolegend, USA) antibody for 15 minutes at 4℃ to determine total amount of leukocytes. Single-cell suspensions were then labeled with fluorescently conjugated anti–mouse antibodies including CD11b-FITC, Gr1-PE/Cy7 and F4/80-APC (all from Biolegend, USA) for 45 minutes at 4℃ to identify neutrophils and macrophages. In addition, anti-mouse CD326-APC and CD74-Alexa Fluor® 488 antibodies (both from Biolegend, USA) were used to identify $CD74^+$ PTECs. The appropriate fluorochrome-conjugated isotype control antibodies were used as negative controls. Cells were then washed and resuspended, and flow cytometry data were acquired on a BD FACSVerse flow cytometer (BD, USA). Further flow cytometry data analysis was performed using Flowjo analysis platform (FlowJo, LLC). Gating strategy are provided in Figure S12.

Reporting checklist for study

We have completed the reporting checklist for study based on the ARRIVE reporting guidelines[12].

Supplementary References

1. Li T, Sun H, Li Y, Su L, Jiang J, Liu Y, et al. Downregulation of macrophage migration inhibitory factor attenuates NLRP3 inflammasome mediated pyroptosis in sepsis-induced AKI. Cell Death Discov. 2022; 8: 61.
2. Liu Y, Liu Y, Wang Q, Song Y, Chen S, Cheng B, et al. MIF inhibitor ISO-1 alleviates severe acute pancreatitis-associated acute kidney injury by suppressing the NLRP3 inflammasome signaling pathway. Int Immunopharmacol. 2021; 96: 107555.
3. Dhillon P, Park J, Hurtado Del Pozo C, Li L, Doke T, Huang S, et al. The Nuclear Receptor ESRRA Protects from Kidney Disease by Coupling Metabolism and Differentiation. Cell Metab. 2021; 33: 379-94.e8.
4. Park J, Shrestha R, Qiu C, Kondo A, Huang S, Werth M, et al. Single-cell transcriptomics of the mouse kidney reveals potential cellular targets of kidney disease. Science. 2018; 360: 758-63.
5. Zimmerman KA, Bentley MR, Lever JM, Li Z, Crossman DK, Song CJ, et al. Single-Cell RNA Sequencing Identifies Candidate Renal Resident Macrophage Gene Expression Signatures across Species. J Am Soc Nephrol. 2019; 30: 767-81.
6. Alshetaiwi H, Pervolarakis N, McIntyre LL, Ma D, Nguyen Q, Rath JA, et al. Defining the emergence of myeloid-derived suppressor cells in breast cancer using single-cell transcriptomics. Sci Immunol. 2020; 5.

7. Kohanbash G, Carrera DA, Shrivastav S, Ahn BJ, Jahan N, Mazor T, et al. Isocitrate dehydrogenase mutations suppress STAT1 and CD8+ T cell accumulation in gliomas. J Clin Invest. 2017; 127: 1425-37.

8. Shan Y, Zhang Y, Zhao Y, Wang T, Zhang J, Yao J, et al. JMJD3 and UTX determine fidelity and lineage specification of human neural progenitor cells. Nat Commun. 2020; 11: 382.

9. Jin S, Guerrero-Juarez CF, Zhang L, Chang I, Ramos R, Kuan CH, et al. Inference and analysis of cell-cell communication using CellChat. Nat Commun. 2021; 12: 1088.

10. Rao Y, Zhong D, Qiu K, Cheng D, Li L, Zhang Y, et al. Single-Cell Transcriptome Profiling Identifies Phagocytosis-Related Dual-Feature Cells in A Model of Acute Otitis Media in Rats. Front Immunol. 2021; 12: 760954.

11. Velasco S, Kedaigle AJ, Simmons SK, Nash A, Rocha M, Quadrato G, et al. Individual brain organoids reproducibly form cell diversity of the human cerebral cortex. Nature. 2019; 570: 523-7.

12. Percie du Sert N, Hurst V, Ahluwalia A, Alam S, Avey MT, Baker M, et al. The ARRIVE guidelines 2.0: Updated guidelines for reporting animal research. PLoS Biol. 2020; 18: e3000410.

Supplementary Tables

Experiment	Antibody	Corp.	Cat. Number	Dilution
immunohistochemistry	AOAH	Abcam	ab222913	1:100
	CD74	Santa Cruz	sc-6262	1:50
immunofluorescence	α-SMA	Abcam	ab124964	1:1000
	CD74	Santa Cruz	sc-6262	1:50
	CD74	Abcam	ab289885	1:100
	LTL	Vector	FL-1321-2	1:500
	NGAL	R&D Systems	AF1857	1:100
	AOAH	Abcam	ab222913	1:100
	Aquaporin1	Abcam	Ab15080	1:200
	Cy™2 AffiniPure Donkey Anti-Rabbit IgG	Jackson ImmunoResearch	711-225-152	1:100
	Alexa Fluor 555-labeled Donkey	Beyotime	A0460	1:500

	Anti-Mouse IgG(H+L)			
western blot	α-SMA	Abcam	ab124964	1:10000
	Fibronectin	Abcam	ab45688	1:1000
	Collagen I	Abcam	ab260043	1:500
	Collagen IV	Abcam	ab6586	1:200
	AOAH	Abcam	ab222913	1:500
	CD74	Santa Cruz	sc-6262	1:200
	β-actin	CST	#8457	1:1000
	GAPDH	CST	#97166	1:1000
	α-tubulin	Proteintech	66031-1-Ig	1:2000
	α-tubulin	CST	#3873	1:1000

Table S1. The antibodies used for immunostaining and western blot analysis.

Gene	Forward primer (5'--3')	Reversed primer (3'--5')
beta-actin	GTGACGTTGACATCCGTAAAGA	GCCGGACTCATCGTACTCC
Aoah	GTTTTCCCAACGCTGCGGGG	TGGCCTTCTGCCCGGGTACA
Ngal	GCAGGTGGTACGTTGTGGG	CTCTTGTAGCTCATAGATGGT

		GC
Il1b	GCAACTGTTCCTGAACTCAACT	ATCTTTTGGGGTCCGTCAACT
Il6	TAGTCCTTCCTACCCCAATTTCC	TTGGTCCTTAGCCACTCCTTC
Tnfa	CCTGTAGCCCACGTCGTAG	GGGAGTAGACAAGGTACAACCC
Cxcl1	CTGGGATTCACCTCAAGAACATC	CAGGGTCAAGGCAAGCCTC
Cxcl2	CCAACCACCAGGCTACAGG	GCGTCACACTCAAGCTCTG
Ccl2	TTAAAAACCTGGATCGGAACCAA	GCATTAGCTTCAGATTTACGGGT
Fn	ATGTGGACCCCTCCTGATAGT	GCCCAGTGATTTCAGCAAAGG
Tgfb2	GCCCAGTGATTTCAGCAAAGG	AGTGGATGGATGGTCCTATTACA
Cd74	CGCGACCTCATCTCTAACCAT	ACAGGTTTGGCAGATTTCGGA
C3	CCAGCTCCCCATTAGCTCTG	GCACTTGCCTCTTTAGGAAGTC
C5	GAACAAACCTACGTCATTTCAGC	GTCAACAGTGCCGCGTTTT

Spp1	AGCAAGAAACTCTTCCAAGC<br>AA	GTGAGATTCGTCAGATTCATC<br>CG
Cd44	TCGATTTGAATGTAACCTGCC<br>G	CAGTCCGGGAGATACTGTAG<br>C

Table S2. The primer sequences used for qRT-PCR.

Supplementary Figures

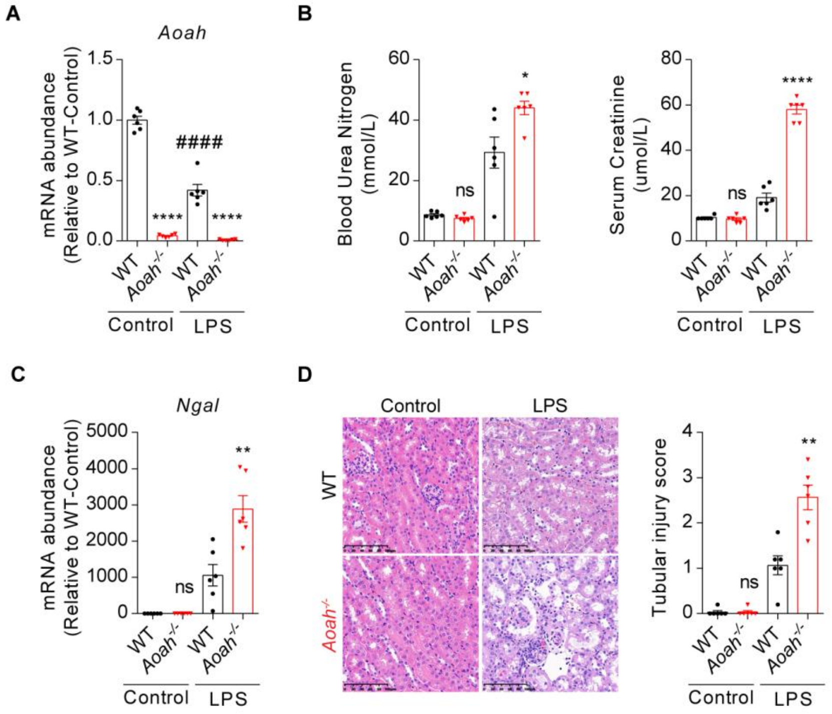

Figure S1. ***Aoah***$^{-/-}$ **mice exhibited more severe kidney injury than WT mice in lipopolysaccharide (LPS)-induced AKI model.** Male WT and *Aoah*$^{-/-}$ mice received LPS (10mg/kg) or vehicle and were sacrificed 24 hours after LPS injection. (A) *Aoah* mRNA expression in kidneys was examined by qPCR. (B) BUN and SCr levels were tested at 24 hours after LPS injection. (C) The mRNA level of *Ngal* after LPS administration. (D) The kidney sections (3μm) were used for hematoxylin-eosin (HE) staining to assess proximal cortical tubular injury. Representative images of HE staining were shown at 400x magnification. Scale bar, 100μm. Renal damage were evaluated semiquantitatively according to the percentage of damaged tubules: 0 = no damage; 1 = 0-25% damaged tubules; 2 = 25-50% damaged tubules; 3 =

50-75% damaged tubules; 4 = >75% damaged tubules. Data were presented as mean ± SEM. Two-tailed student's t test was used to calculate statistical significance. * represents comparison between WT and *Aoah*$^{-/-}$ mice; # represents comparison within WT mice groups; *P<0.05, **P<0.01, ****P<0.0001; ####P<0.0001; ns indicates no significant differences; n=6 for all. Similar results were obtained in 3 independent experiments with 6 to 7 mice per group.

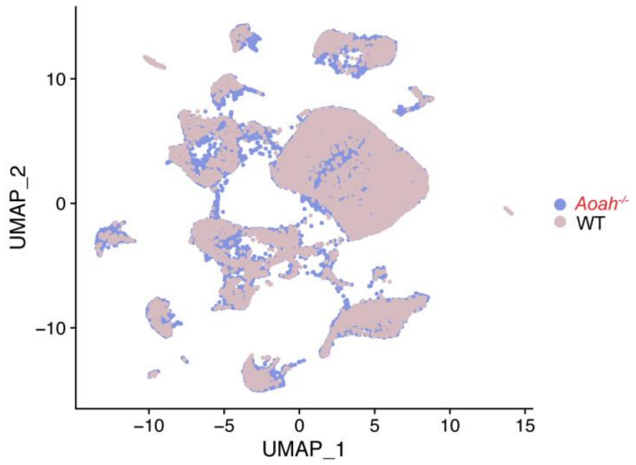

Figure S2. The consistency of sequencing samples between *Aoah*$^{-/-}$ mice and WT mice was assessed by uniform manifold approximation and projection for dimension reduction (UMAP) diagram.

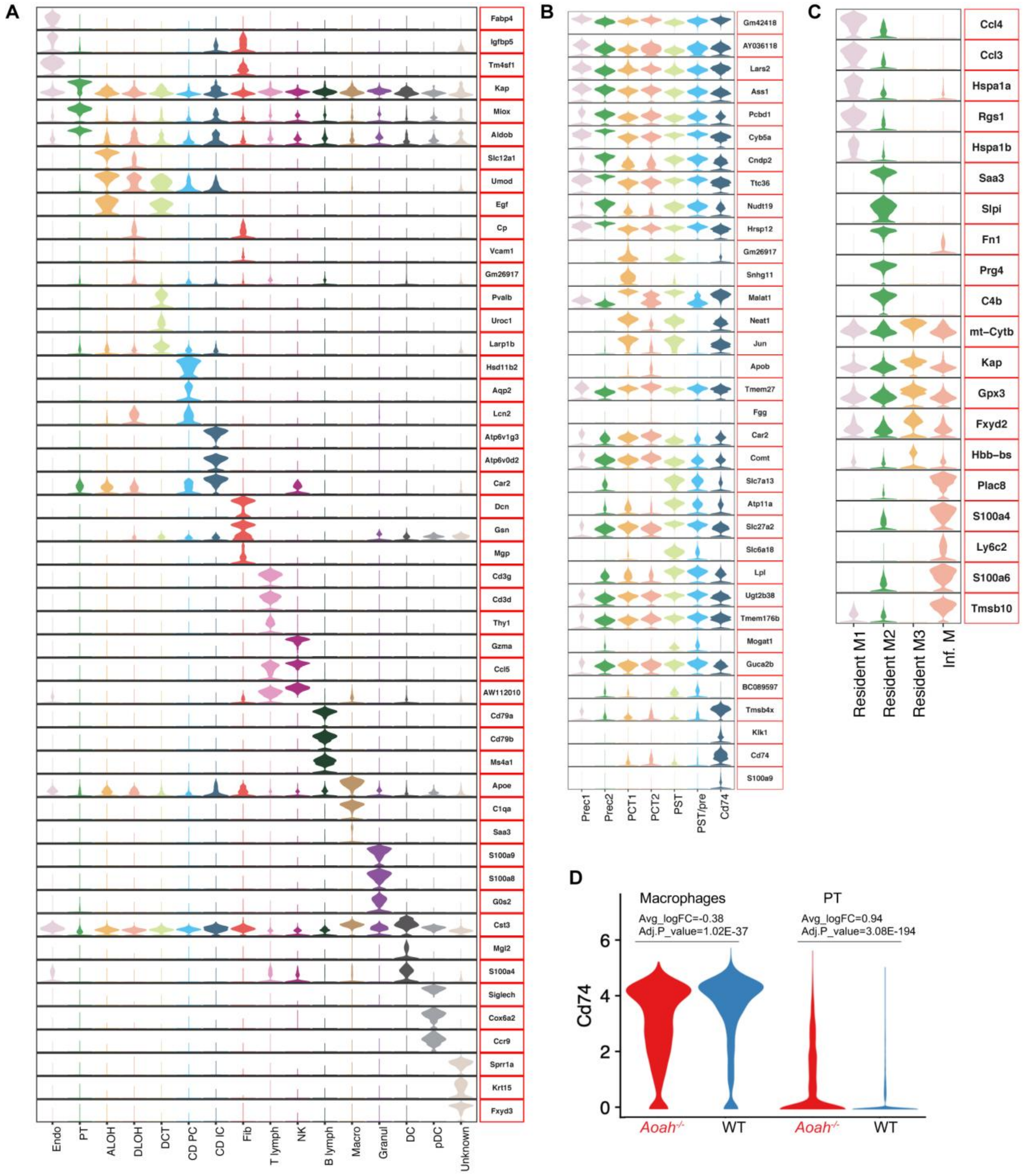

Figure S3. Cell markers used for clustering and classification of cell populations. CD74 expression levels in macrophages and PT cells. (A) The main cell markers used to identify 16 mouse kidney cell types. (B) The main cell markers used to identify PTECs subsets. (C) The main cell markers used to identify macrophages subsets. (D) Differences of *Cd74* mRNA level in macrophages and PTECs between *Aoah*$^{-/-}$ mice and WT mice were inferred by scRNA-seq.

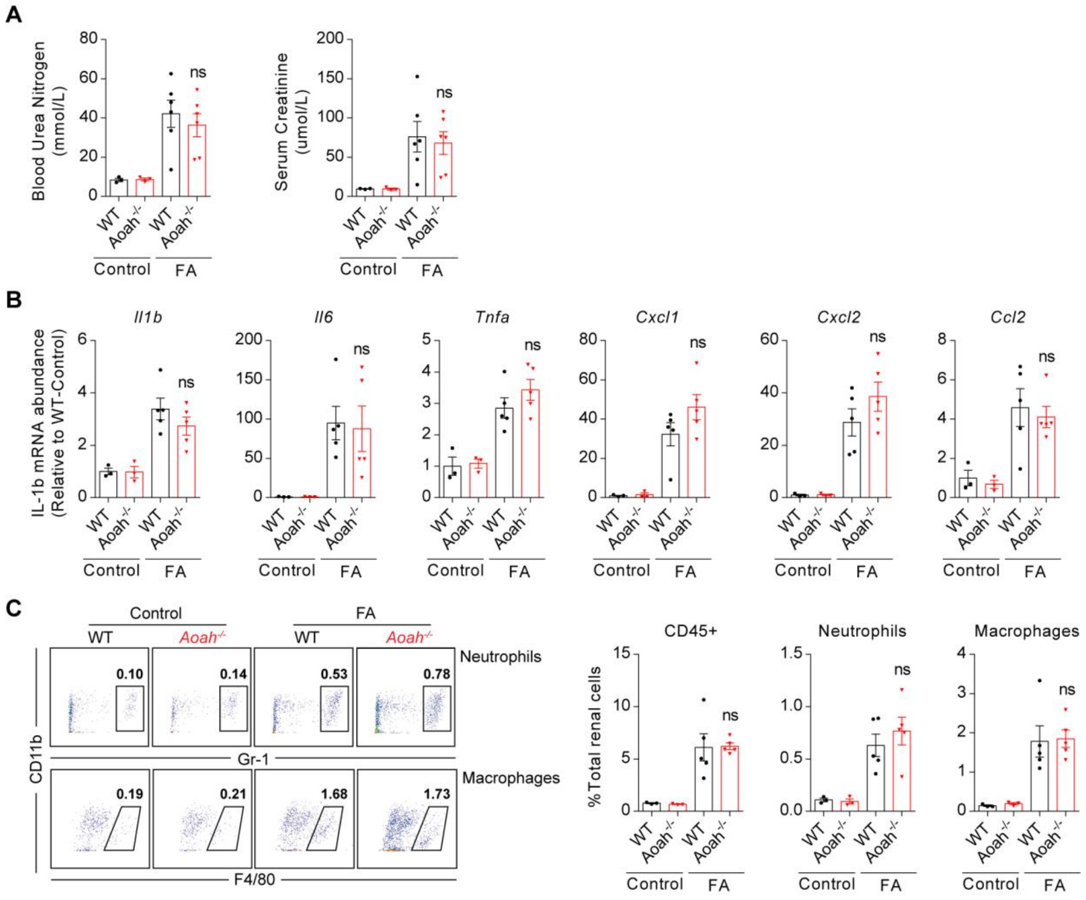

Figure S4. The inflammatory responses were comparable in WT and *Aoah*$^{-/-}$ mice at an early stage of FA-induced renal fibrosis model. Male WT and *Aoah*$^{-/-}$ mice aged 8-10 weeks intraperitoneally received FA (250 mg/kg) or vehicle and were euthanized 24 hours later. (A) BUN and SCr levels were examined at 24 hours after FA injection. (B) The mRNA level of pro-inflammatory cytokines between WT and *Aoah*$^{-/-}$ mice at 24 hours after FA injection was determined by qPCR. (C) Proportion of total leukocytes, neutrophils and macrophages in kidney-derived single-cell suspensions. CD45^{+} cells were considered total leukocytes; CD11b^{+}Gr-1^{+} cells were considered neutrophils; CD11b^{+}F4/80^{+} cells were considered macrophages.

Data were presented as mean ± SEM. Two-tailed student's t test was used to calculate statistical significance. ns indicates no significant differences; *n*=6 for all. Similar results were obtained in 3 independent experiments with 6 to 7 mice per group.

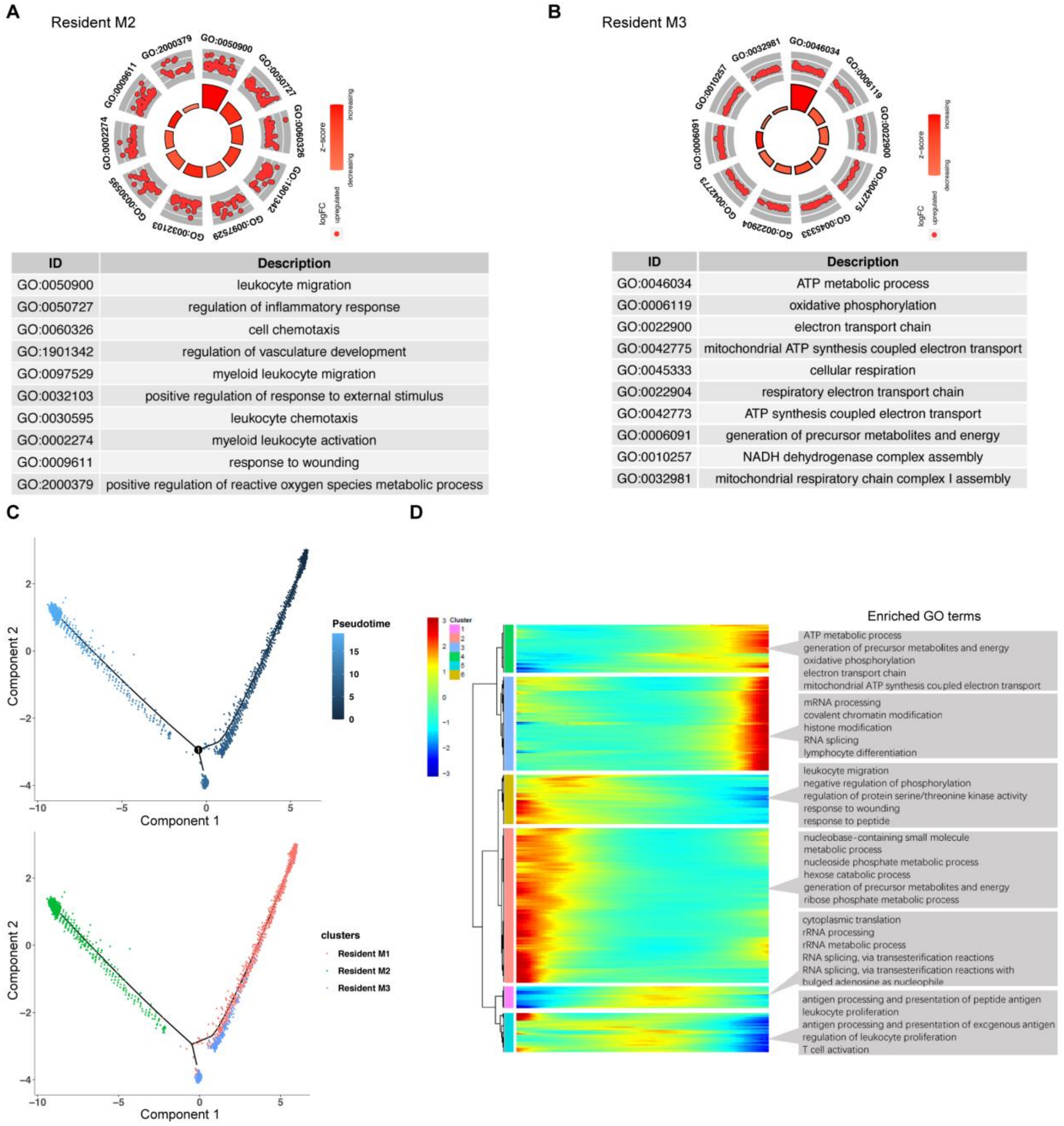

Figure S5. Function analysis and pseudo-time analysis of macrophages subsets. (A) GO analysis was used for resident M2 macrophages, showing enrichment of terms related to biological processes, such as leukocyte

migration and regulation of inflammatory response. (B) GO analysis was used for resident M3 macrophages, showing enrichment of terms related to biological processes, such as ATP metabolic process and oxidative phosphorylation. (C) Pseudo-time analysis was used to infer differentiation trajectories of resident macrophages. (D) GO analysis showing enriched terms related to the differentiation conversion of resident macrophages.

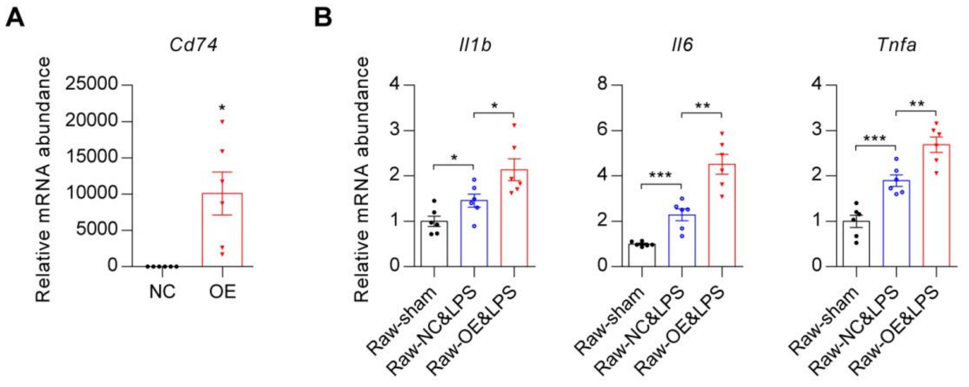

Figure S6. ***Cd74*****-overexpressing mouse tubular epithelial cells (mTECs) promoted macrophages M1 polarization.** (A) Overexpression plasmids were used to transfect mTECs. Transfection efficiency of *Cd74* overexpression plasmids or control plasmids was determined by qPCR. NC: control plasmid group; OE: *Cd74*-overexpressing group. (B) RAW 264.7 macrophages were cultured alone without intervention, or co-cultured with *Cd74*-overexpressing mTECs or control mTECs with LPS stimulation. RAW 264.7 cells were collected 12 hours later. The mRNA level of *Il1β*, *Il6* and *Tnfα* was measured by qPCR. Data were presented as mean ± SEM. Two-tailed student's t test was used to calculate statistical significance. * represents comparison

between two designated groups; *P<0.05, **P<0.01, ****P<0.0001; n=6 for all. Similar results were obtained from 3 independent experiments.

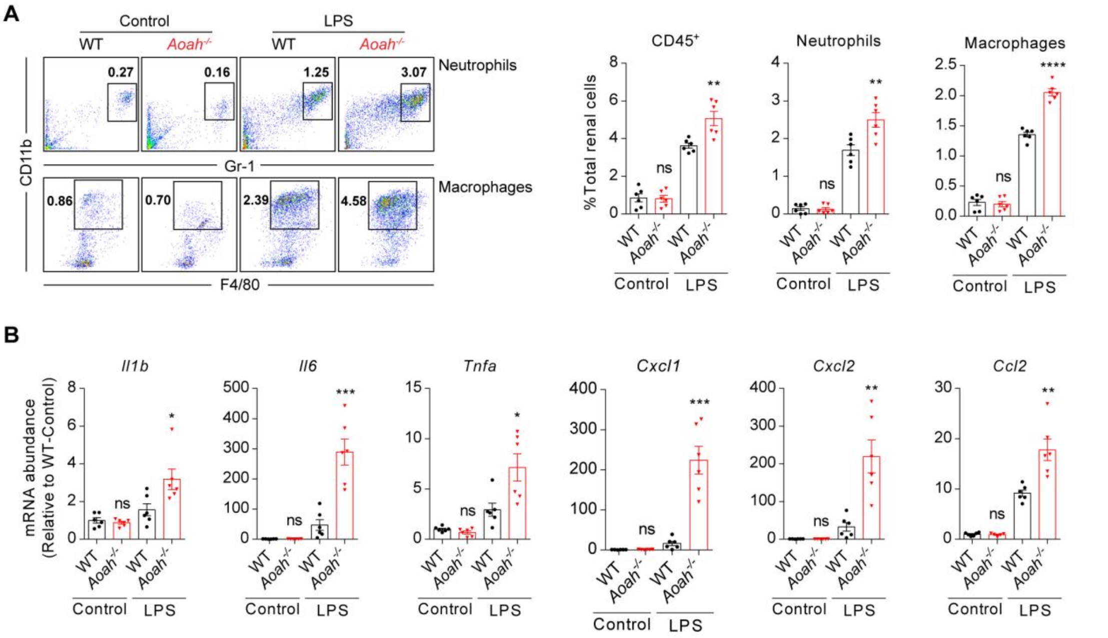

Figure S7. *Aoah* deficiency promoted the recruitment of innate immune cells and the production of inflammatory cytokines in LPS-induced AKI model. Male WT and *Aoah*$^{-/-}$ mice aged 8-10 weeks intraperitoneally received FA (250 mg/kg) or vehicle and were euthanized 2 weeks later. (A) Proportion of total leukocytes, neutrophils and macrophages in kidney-derived single-cell suspensions. CD45^{+} cells were considered total leukocytes; CD11b^{+}Gr-1^{+} cells were considered neutrophils; CD11b^{+}F4/80^{+} cells were considered macrophages. (B) The mRNA level of inflammatory cytokines, including *Il1b*, *Il6*, *Tnfa*, *Cxcl1*, *Cxcl2*, *Ccl2*, were determined by qPCR. Data were presented as mean ± SEM. Two-tailed student's t test was used to calculate statistical significance. * represents comparison between WT and *Aoah*$^{-/-}$ mice; *P<0.05,

**P<0.01, ***P<0.001, ****P<0.0001; ns indicates no significant differences; *n*=6 for all. Similar results were obtained in 3 independent experiments with 6 to 7 mice per group.

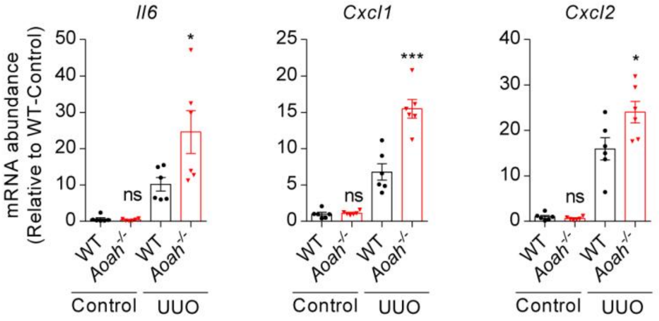

Figure S8. *Aoah* deficiency promoted the production of inflammatory cytokines in UUO model. Male WT and *Aoah*$^{-/-}$ mice aged 8-10 weeks were sham-operated or underwent ureter ligation and were euthanized 2 weeks later. The mRNA levels of *Il6*, *Cxcl1* and *Cxcl2* were examined by qPCR. Data were presented as mean ± SEM. Two-tailed student's t test was used to calculate statistical significance. * represents comparison between WT and *Aoah*$^{-/-}$ mice; *P<0.05, ***P<0.001; ns indicates no significant differences; *n*=6 for all. Similar results were obtained in 3 independent experiments with 6 to 7 mice per group.

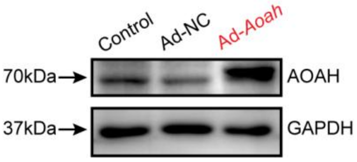

Figure S9. The *Aoah*-overexpressing HEK-293T cells were established and *Aoah* expression was verified by western blot analysis. Western blot showing successful AOAH protein overexpression.

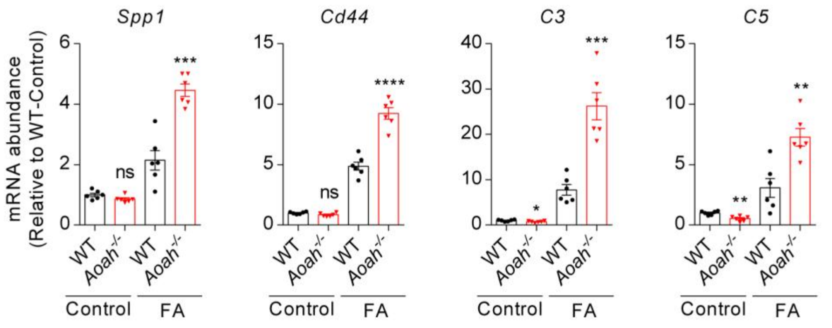

Figure S10. The qPCR analysis confirmed that mRNA expression of *Spp1*, *Cd44*, *C3* and *C5* significantly increased in *Aoah*$^{-/-}$ mice in FA model. Male WT and *Aoah*$^{-/-}$ mice aged 8-10 weeks intraperitoneally received FA (250 mg/kg) or vehicle and were euthanized 2 weeks later. Increased mRNA expression levels of some ligands were verified by qPCR. Data were presented as mean ± SEM. Two-tailed student's t test was used to calculate statistical significance. * represents comparison between WT and *Aoah*$^{-/-}$ mice; $^{**}P<0.01$, $^{***}P<0.001$, $^{****}P<0.0001$; ns indicates no significant differences; $n=6$ for all. Similar results were obtained in 3 independent experiments with 6 to 7 mice per group.

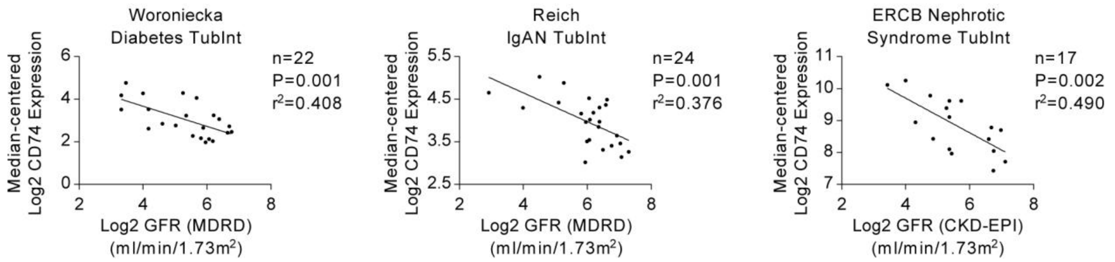

Figure S11. The correlation between CD74 expression and eGFR from the Nephroseq database. CD74 expression level was negatively correlated with

eGFR in CKD patients. CD74 expression data were obtained from the datasets: ‘Woroniecka Diabetes TubInt’, ‘Reich IgAN TubInt’ and ‘ERCB Nephrotic Syndrome TubInt’.

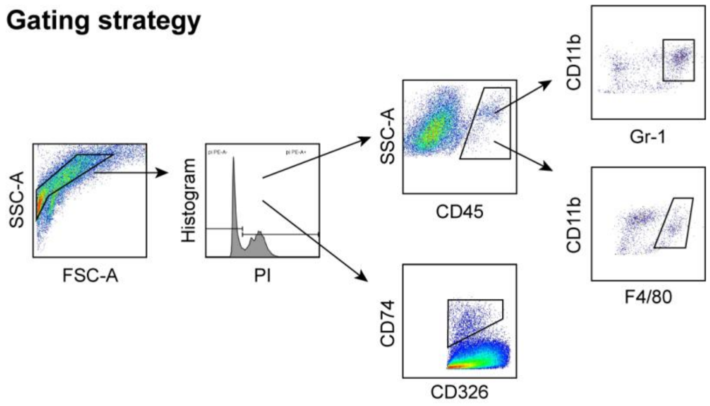

Figure S12. The gating strategy for flow cytometry.

Abbreviations

IACUC: Institutional Animal Care and Use Committee; FA: folic acid; UUO: unilateral ureteral obstruction; LPS: lipopolysaccharide; mTEC: mouse tubular epithelial cell; scRNA-seq: single-cell RNA sequencing; UMI: unique molecular identifier; GEM: Gel Beads-in-Emulsion; UMAP: Uniform Manifold Approximation and Projection; GO: Gene Ontology; HE: hematoxylin-eosin.

Western Blot Origin Data

Figure 1A. Full length membrane

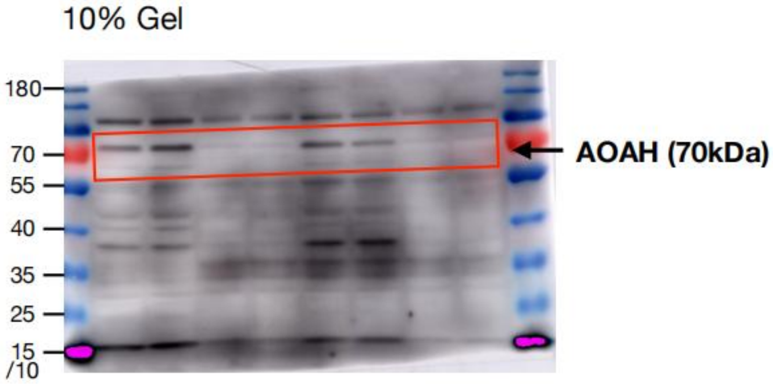

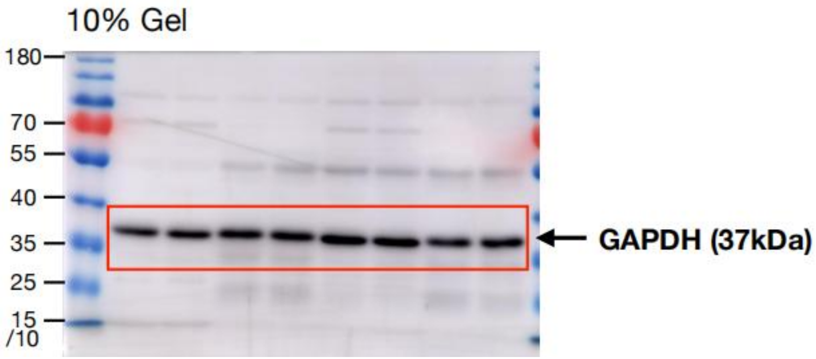

Figure 1F. Full length membrane

12% Gel

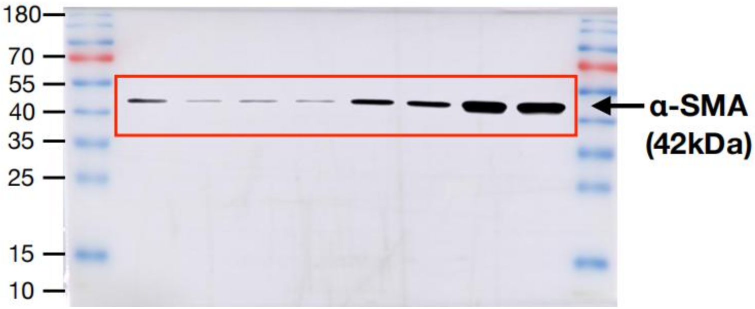

12% Gel

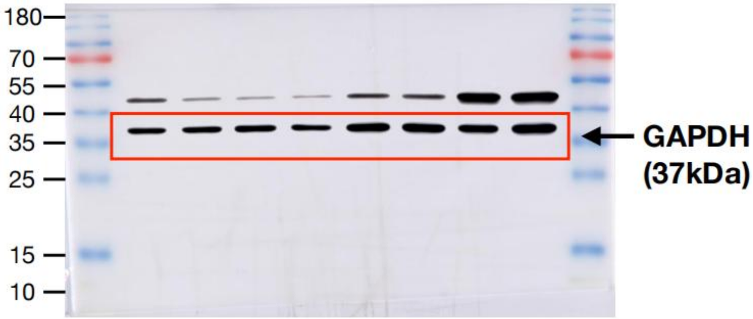

6% Gel

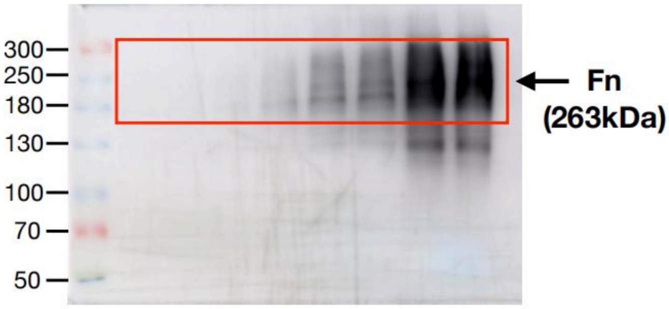

6% Gel

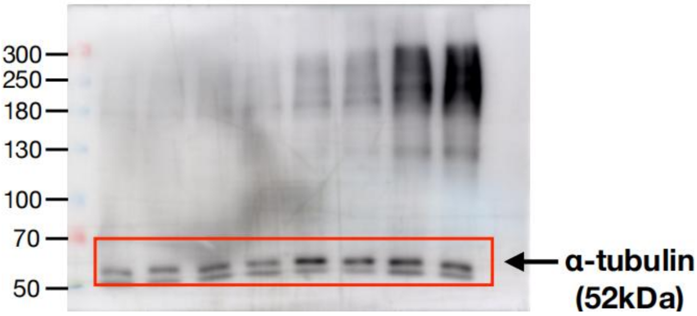

6% Gel

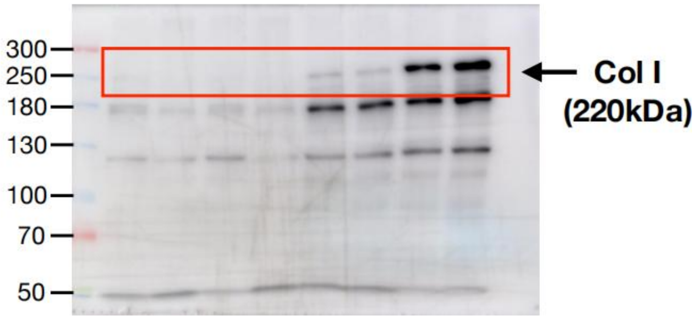

6% Gel

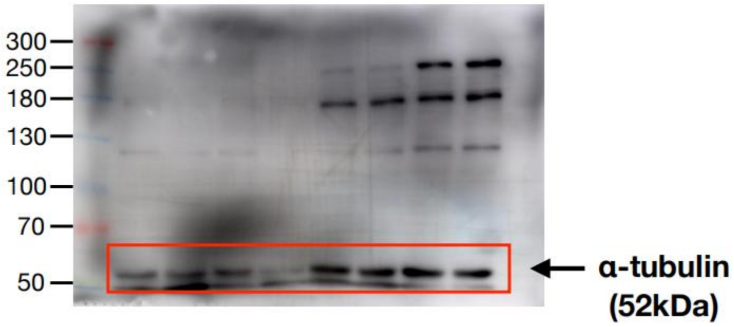

6% Gel

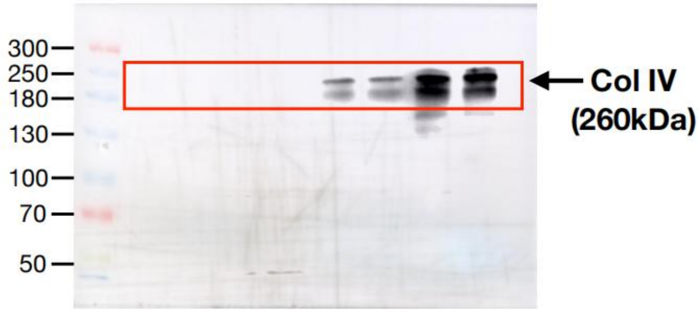

6% Gel

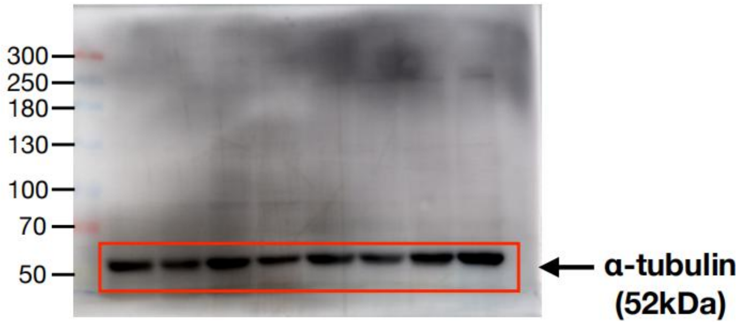

Figure 2D. Full length membrane

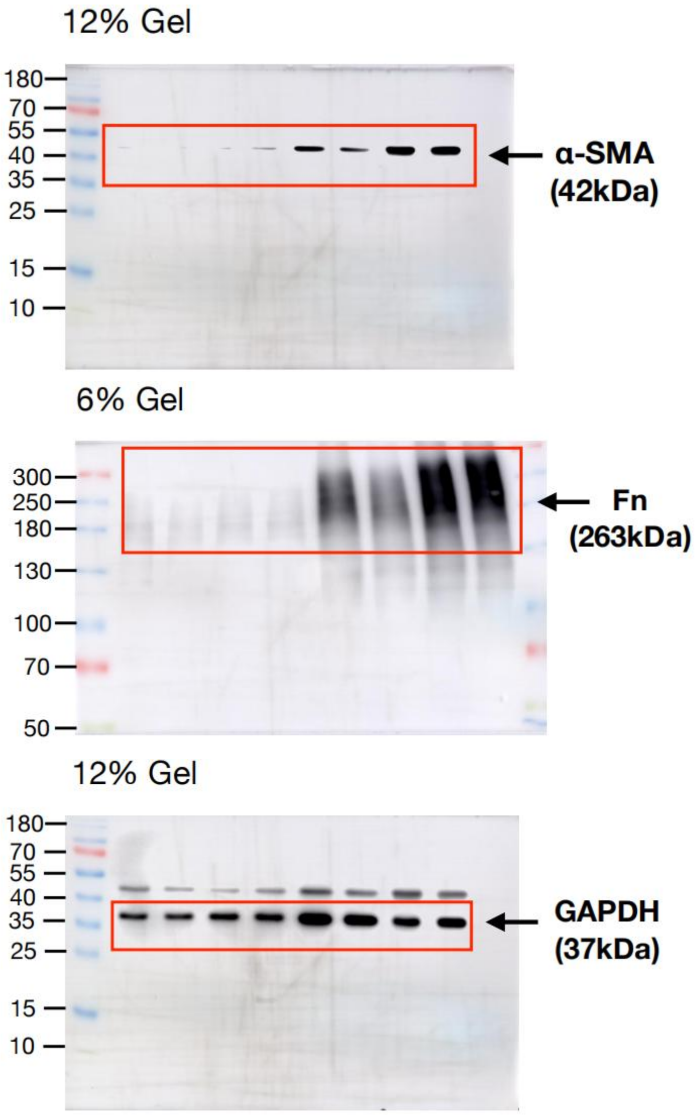

6% Gel

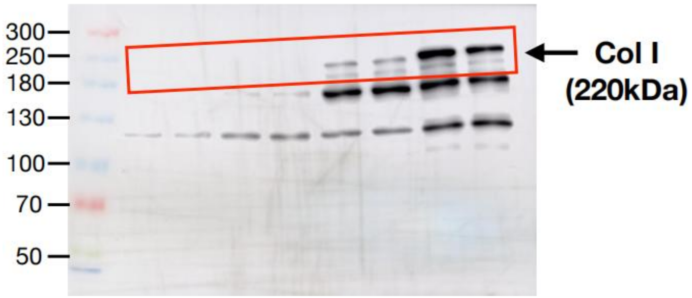

6% Gel

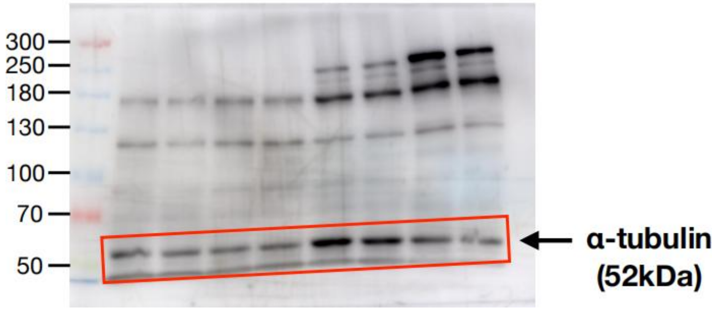

6% Gel

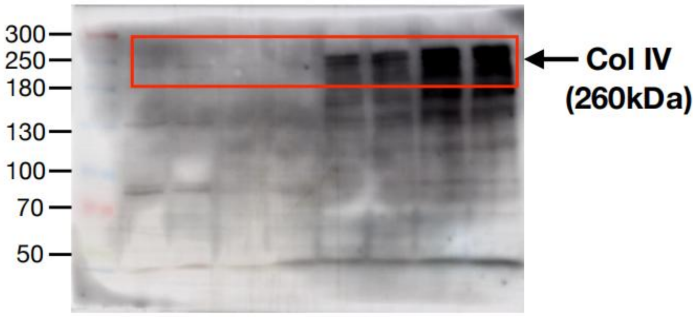

6% Gel

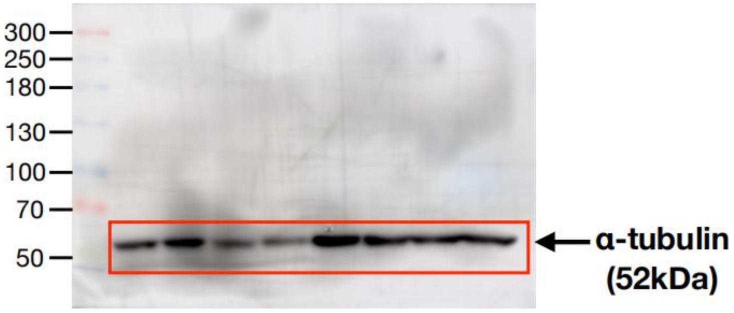

Figure 4A. Full length membrane

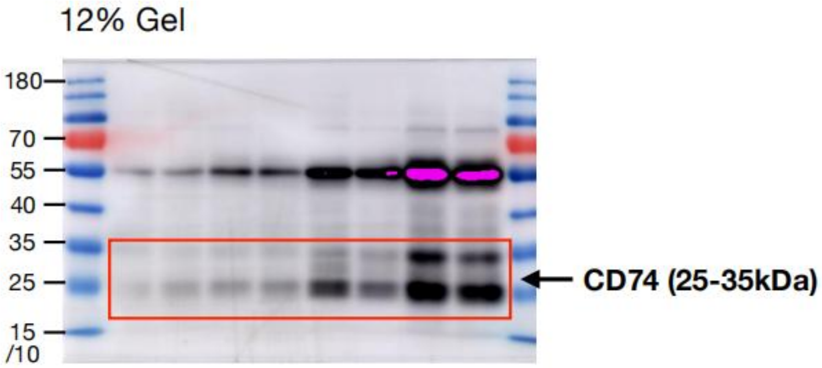

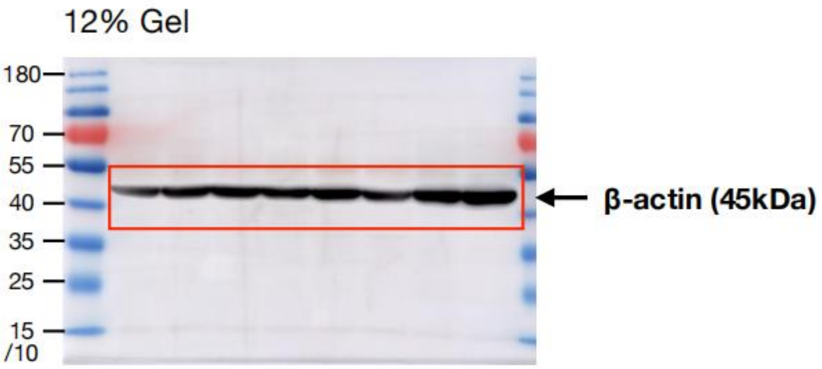

Figure 6C. Full length membrane

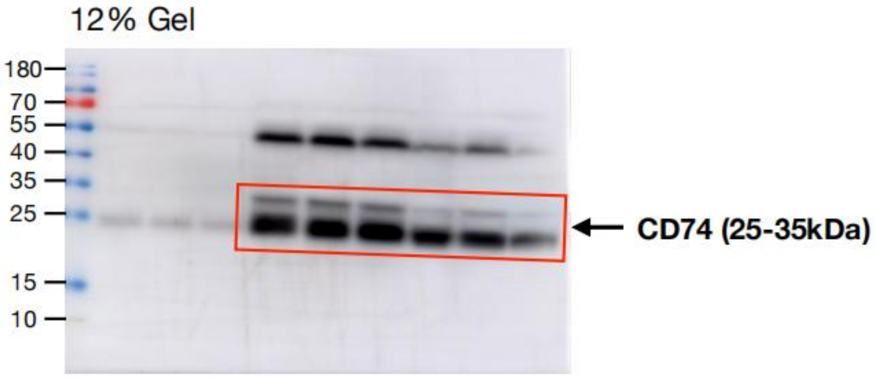

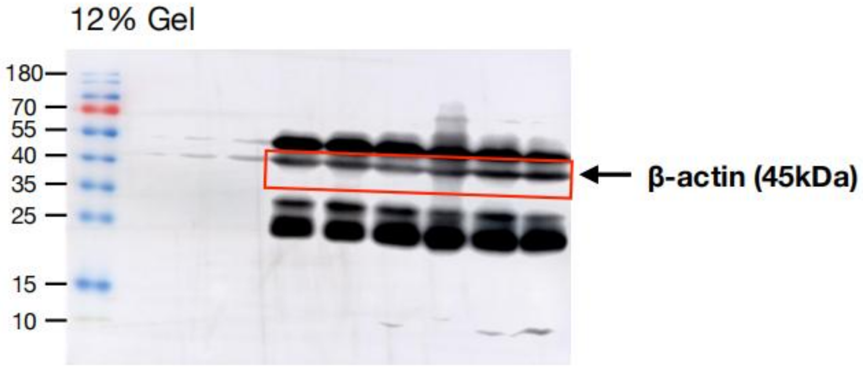

Figure 6G. Full length membrane

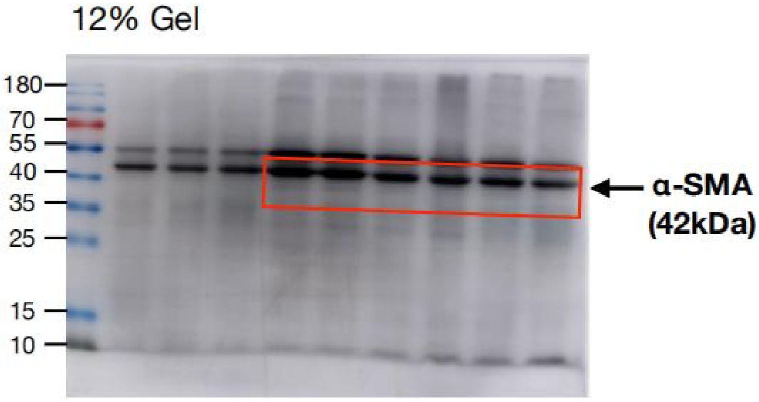

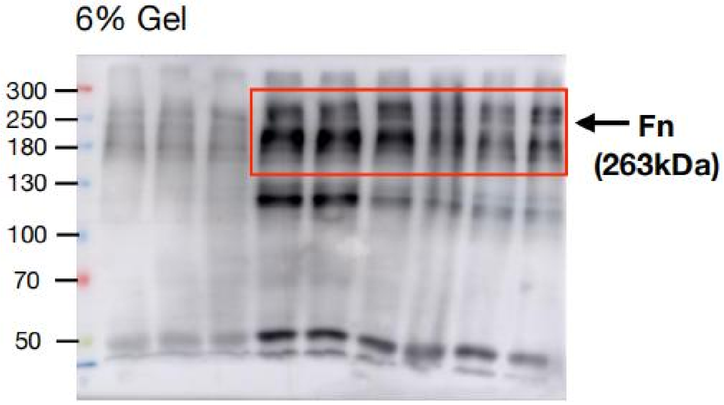

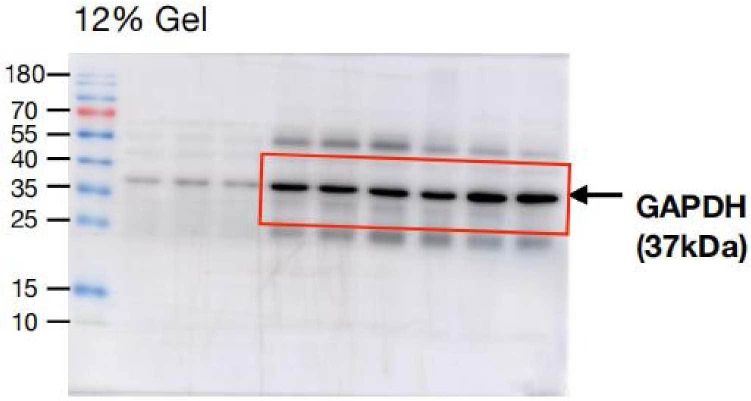

6% Gel

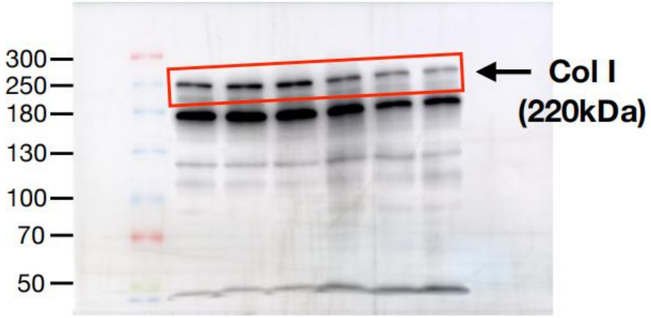

6% Gel

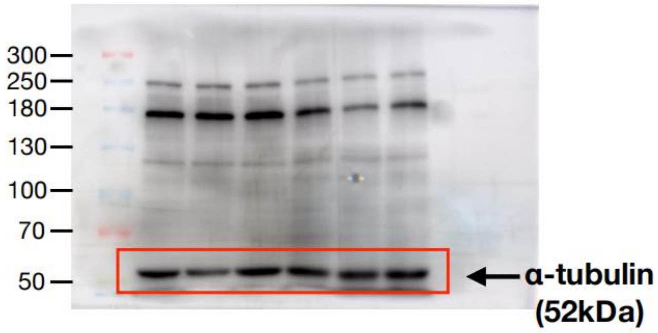

6% Gel

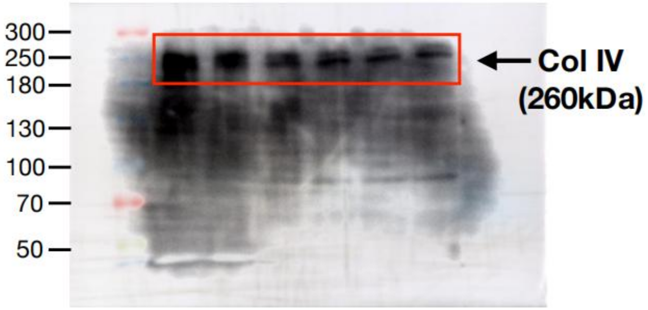

6% Gel

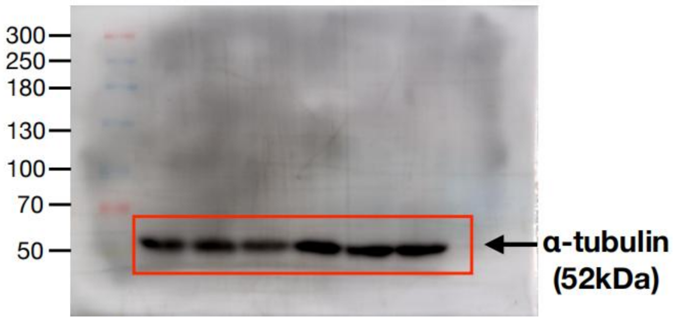

Figure 8D. Full length membrane

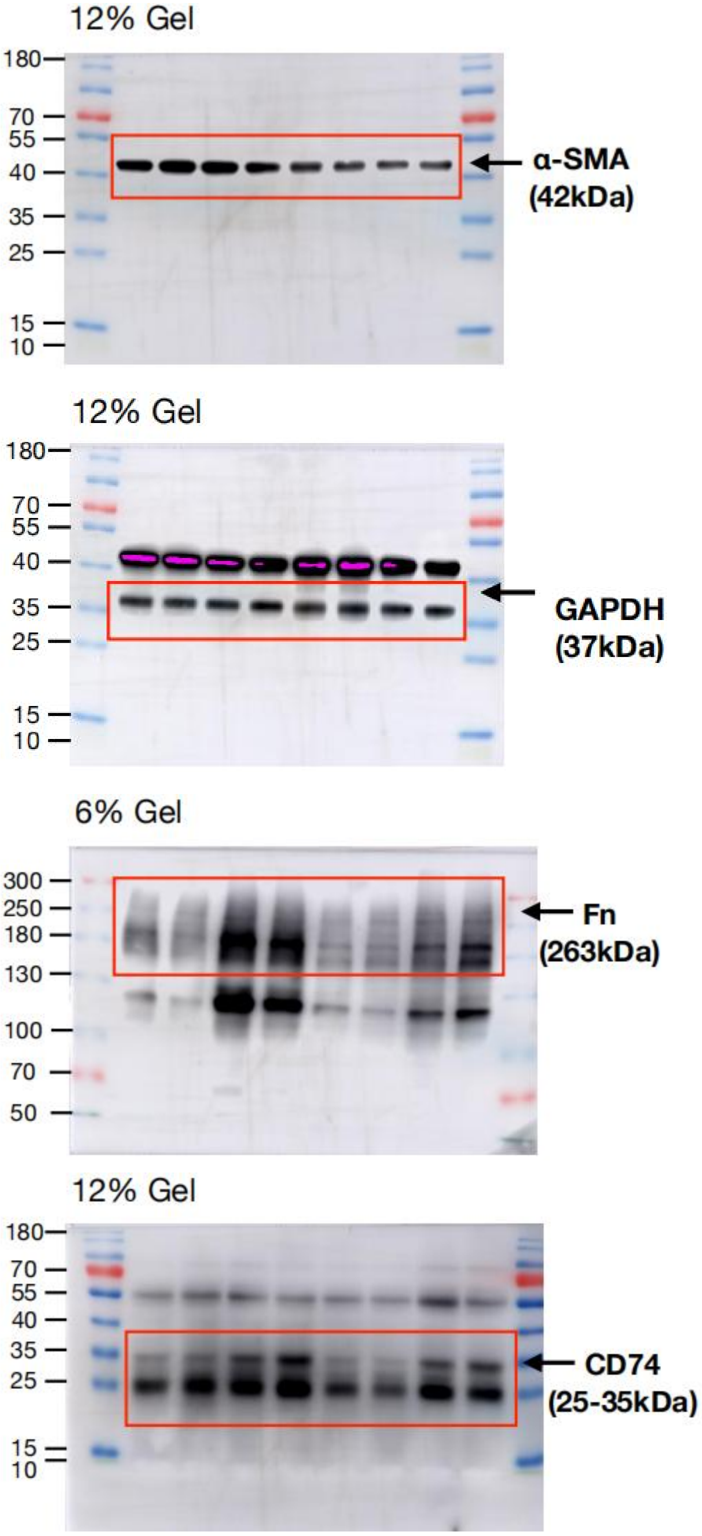

6% Gel

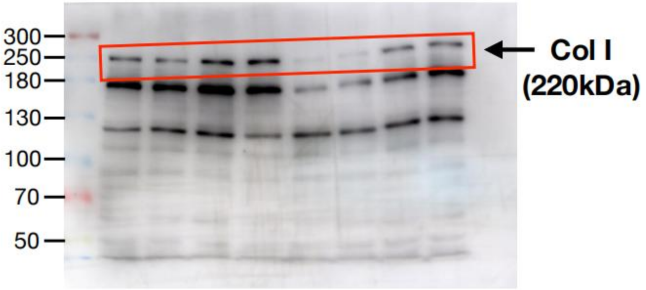

6% Gel

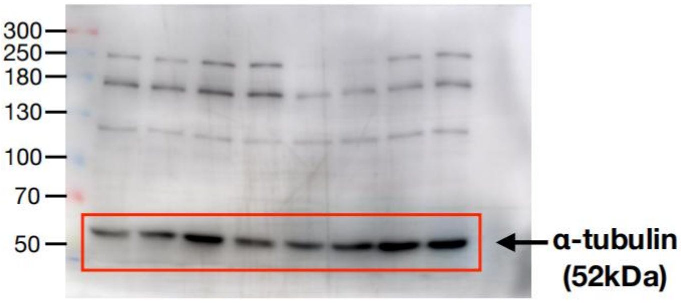

6% Gel

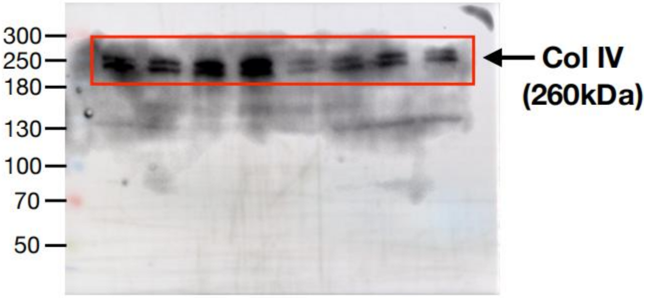

6% Gel

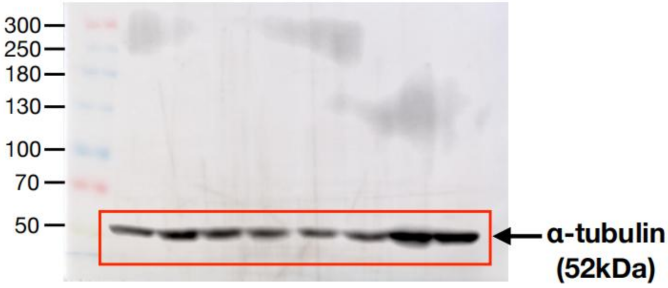

Figure S9. Full length membrane

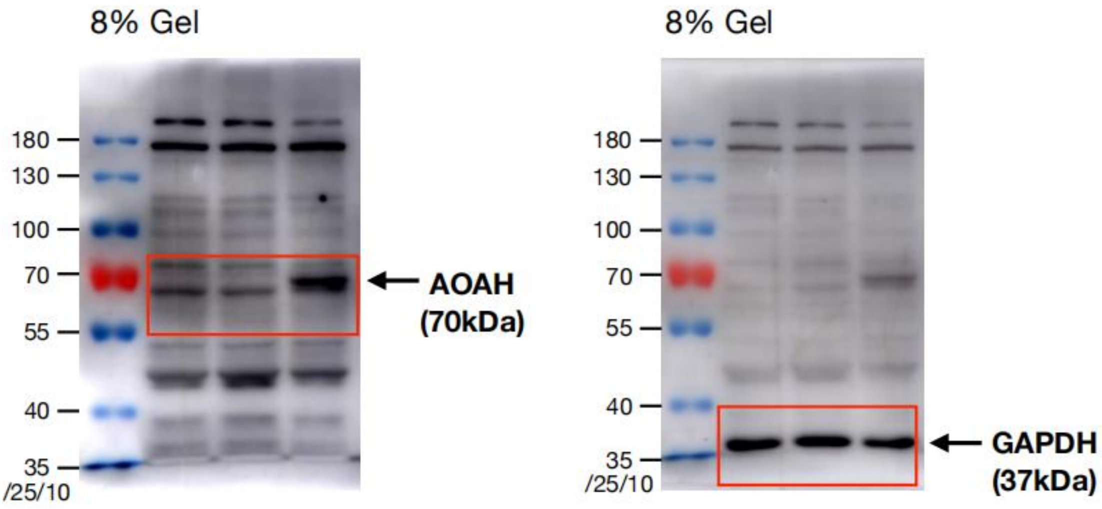

Reporting checklist for study using laboratory animals.

Based on the ARRIVE guidelines.

Instructions to authors

Complete this checklist by entering the page numbers from your manuscript where readers will find each of the items listed below.
Your article may not currently address all the items on the checklist. Please modify your text to include the missing information. If you are certain that an item does not apply, please write "n/a" and provide a short explanation.
Upload your completed checklist as an extra file when you submit to a journal.
In your methods section, say that you used the ARRIVEreporting guidelines, and cite them as:
Percie du Sert N, Hurst V, Ahluwalia A, Alam S, Avey MT, Baker M, Browne WJ, Clark A, Cuthill IC, Dirnagl U, Emerson M, Garner P, Holgate ST, Howells DW, Karp NA, Lazic SE, Lidster K, MacCallum CJ, Macleod M, Pearl EJ, Petersen O, Rawle F, Peynolds P, Rooney K, Sena ES, Silberberg SD, Steckler T and Wurbel H. The ARRIVE Guidelines 2.0: updated guidelines for reporting animal research.

		Reporting Item	Page Number
**Essential 10**			
Study design	#1a	Give details of the groups being compared, including control groups. If no control group has been used, the rationale should be stated.	52-53, see Supplementary Methods - 'Animals and models'
Study design	#1b	Give details of the experimental unit (e.g., a single animal, litter, or cage of animals).	36-51, 70-79, see 'Figure legends'
Sample size	#2a	Specify the exact number of experimental units allocated to each group, and the total number in each experiment. Also indicate the total number of animals used.	36-51, 70-79, see 'Figure legends'
Sample size	#2b	Explain how the sample size was decided. Provide details of any a priori sample size calculation, if done.	n/a; sample size was determined based on previous experience with the animal model in our laboratory; a priori sample size calculations were not conducted
Inclusion and exclusion criteria	#3a	Describe any criteria used for including or excluding animals (or experimental units) during the experiment, and data points during the analysis. Specify if these criteria were established a priori. If	n/a; no criteria were used for including and excluding animals during the experiment

		no criteria were set, state this explicitly.	
Inclusion and exclusion criteria	#3b	For each experimental group, report any animals, experimental units, or data points not included in the analysis and explain why. If there were no exclusions, state so.	n/a; all animals reported, except that 1 Aoah-/- mouse died during ISO-1 treatment experiment
Inclusion and exclusion criteria	#3c	For each analysis, report the exact value of n in each experimental group.	36-51, 70-79, see 'Figure legends'
Randomisation	#4a	State whether randomisation was used to allocate experimental units to control and treatment groups. If done, provide the method used to generate the randomisation sequence.	52-53, by cage
Randomisation	#4b	Describe the strategy used to minimise potential confounders such as the order of treatments and measurements, or animal/cage location. If confounders were not controlled, state this explicitly.	52-55, see Supplementary Methods
Blinding	#5	Describe who was aware of the group allocation at the different stages of the experiment (during the allocation, the conduct of the experiment, the outcome assessment, and the data analysis).	59-61, the stained kidney sections were evaluated in a double-blind fashion
Outcome measures	#6a	Clearly define all outcome measures assessed (e.g., cell death, molecular markers, or behavioural changes).	36-51, 70-79, see 'Figure legends'
Outcome measures	#6b	For hypothesis-testing studies, specify the primary outcome measure, i.e., the outcome measure that was used to determine the sample size.	n/a
Statistical methods	#7a	Provide details of the statistical methods	9, see Methods - Statistics

		used for each analysis, including software used.	
Statistical methods	#7b	Describe any methods used to assess whether the data met the assumptions of the statistical approach, and what was done if the assumptions were not met.	n/a
Experimental animals	#8a	Provide species-appropriate details of the animals used, including species, strain and substrain, sex, age or developmental stage, and, if relevant, weight.	8, 36-51, 70-79, see 'Figure legends'
Experimental animals	#8b	Provide further relevant information on the provenance of animals, health/immune status, genetic modification status, genotype, and any previous procedures.	8, see 'Methods-Animals and models'
Experimental procedures	#9a	For each experimental group, including controls, describe the procedures in enough detail to allow others to replicate what was done, how it was done, and what was used.	52-63, see Supplementary Materials
Experimental procedures	#9b	Timing and frequency of procedures	52-63, see Supplementary Materials
Experimental procedures	#9c	Where procedures were carried out (including detail of any acclimatisation periods).	52-63, see Supplementary Materials
Experimental procedures	#9d	Rationale for procedures	10-24, see 'Results'
Results	#10a	For each experiment conducted, including independent replications, report summary/descriptive statistics for each experimental group, with a measure of variability where applicable (e.g., mean and SD, or median and range).	

36-51, 70-79, see 'Figure legends'			
Results	#10b	If applicable, for each experiment conducted, including independent replications, report the effect size with a confidence interval.	n/a
**Recommended set**			
Abstract	#11	Provide an accurate summary of the research objectives, animal species, strain and sex, key methods, principal findings, and study conclusions.	3-4, see 'Abstract'
Background	#12a	Include sufficient scientific background to understand the rationale and context for the study, and explain the experimental approach.	5-7, see 'Introduction'
Background	#12b	Explain how the animal species and model used address the scientific objectives and, where appropriate, the relevance to human biology.	5-7, see 'Introduction'
Objectives	#13	Clearly describe the research question, research objectives and, where appropriate, specific hypotheses being tested.	6, 20, see 'Introduction' and 'Discussion'
Ethical statement	#14	Provide the name of the ethical review committee or equivalent that has approved the use of animals in this study and any relevant licence or protocol numbers (if applicable). If ethical approval was not sought or granted, provide a justification.	8, see 'Methods'
Housing and husbandry	#15	Provide details of housing and husbandry conditions, including any environmental enrichment.	52, see Supplementary Methods - 'Animals and models'
Animal care and monitoring	#16a	Describe any interventions or steps taken in the experimental protocols	52-53, see Supplementary Methods - 'Animals and models'

		to reduce pain, suffering, and distress.	
Animal care and monitoring	#16b	Report any expected or unexpected adverse events.	n/a
Animal care and monitoring	#16c	Describe the humane endpoints established for the study, the signs that were monitored, and the frequency of monitoring. If the study did not set humane endpoints, state this.	n/a; no humane endpoints were utilized for the mice in this study
Interpretation/scientific implications	#17a	Interpret the results, taking into account the study objectives and hypotheses, current theory, and other relevant studies in the literature.	10-19, see 'Results'
Interpretation/scientific implications	#17b	Comment on the study limitations, including potential sources of bias, limitations of the animal model, and imprecision associated with the results.	20-24, see 'Discussion'
Generalisability/translation	#18	Comment on whether, and how, the findings of this study are likely to generalise to other species or experimental conditions, including any relevance to human biology (where appropriate).	20-24, see 'Discussion'
Protocol registration	#19	Provide a statement indicating whether a protocol (including the research question, key design features, and analysis plan) was prepared before the study, and if and where this protocol was registered.	n/a
Data access	#20	Provide a statement describing if and where study data are available.	26, see 'Data availability'
Declaration of interests	#21a	Declare any potential conflicts of interest, including financial and nonfinancial. If none exist, this should be stated.	28, see 'Competing interests'
Declaration of interests	#21b	List all funding sources	27-28, see 'Funding' and 'Author contributions'

(including grant identifier) and the role of the funder(s) in the design, analysis, and reporting of the study.

Notes:

- 1a: 52-53, see Supplementary Methods - 'Animals and models'
- 1b: 36-51, 70-79, see 'Figure legends'
- 2a: 36-51, 70-79, see 'Figure legends'
- 2b: n/a; sample size was determined based on previous experience with the animal model in our laboratory; a priori sample size calculations were not conducted
- 3a: n/a; no criteria were used for including and excluding animals during the experiment
- 3b: n/a; all animals reported, except that 1 Aoah-/- mouse died during ISO-1 treatment experiment
- 3c: 36-51, 70-79, see 'Figure legends'
- 4a: 52-53, by cage
- 4b: 52-55, see Supplementary Methods
- 5: 59-61, the stained kidney sections were evaluated in a double-blind fashion
- 6a: 36-51, 70-79, see 'Figure legends'
- 7a: 9, see Methods - Statistics
- 8a: 8, 36-51, 70-79, see 'Figure legends'
- 8b: 8, see 'Methods-Animals and models'
- 9a: 52-63, see Supplementary Materials
- 9b: 52-63, see Supplementary Materials
- 9c: 52-63, see Supplementary Materials
- 9d: 10-24, see 'Results'
- 10a: 36-51, 70-79, see 'Figure legends'
- 11: 3-4, see 'Abstract'
- 12a: 5-7, see 'Introduction'
- 12b: 5-7, see 'Introduction'
- 13: 6, 20, see 'Introduction' and 'Discussion'
- 14: 8, see 'Methods'
- 15: 52, see Supplementary Methods - 'Animals and models'
- 16a: 52-53, see Supplementary Methods - 'Animals and models'
- 16c: n/a; no humane endpoints were utilized for the mice in this study
- 17a: 10-19, see 'Results'
- 17b: 20-24, see 'Discussion'
- 18: 20-24, see 'Discussion'
- 20: 26, see 'Data availability'
- 21a: 28, see 'Competing interests'
- 21b: 27-28, see 'Funding' and 'Author contributions' The ARRIVE checklist is distributed under the terms of the Creative Commons Attribution License CC-BY. This checklist was completed on 16. May 2024 using https://www.goodreports.org/, a tool made by the EQUATOR Network in collaboration with Penelope.ai
